# Genetic Code Expansion Approaches to Decipher the Ubiquitin Code

**DOI:** 10.1021/acs.chemrev.4c00375

**Published:** 2024-09-23

**Authors:** Vera Wanka, Maximilian Fottner, Marko Cigler, Kathrin Lang

**Affiliations:** † Laboratory for Organic Chemistry (LOC), Department of Chemistry and Applied Biosciences (D-CHAB), ETH Zurich, Vladimir-Prelog-Weg 3, 8093 Zurich, Switzerland; ‡ Department of Chemistry, Technical University of Munich, 85748 Garching, Germany

## Abstract

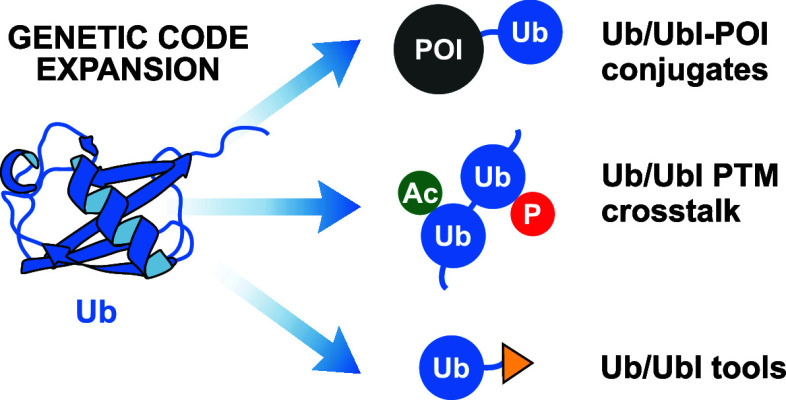

The covalent attachment of Ub (ubiquitin) to target proteins (ubiquitylation) represents one of the most versatile PTMs (post-translational modifications) in eukaryotic cells. Substrate modifications range from a single Ub moiety being attached to a target protein to complex Ub chains that can also contain Ubls (Ub-like proteins). Ubiquitylation plays pivotal roles in most aspects of eukaryotic biology, and cells dedicate an orchestrated arsenal of enzymes to install, translate, and reverse these modifications. The entirety of this complex system is coined the Ub code. Deciphering the Ub code is challenging due to the difficulty in reconstituting enzymatic machineries and generating defined Ub/Ubl–protein conjugates. This Review provides a comprehensive overview of recent advances in using GCE (genetic code expansion) techniques to study the Ub code. We highlight strategies to site-specifically ubiquitylate target proteins and discuss their advantages and disadvantages, as well as their various applications. Additionally, we review the potential of small chemical PTMs targeting Ub/Ubls and present GCE-based approaches to study this additional layer of complexity. Furthermore, we explore methods that rely on GCE to develop tools to probe interactors of the Ub system and offer insights into how future GCE-based tools could help unravel the complexity of the Ub code.

## 1 Introduction

### 1.1 The Ubiquitin System: Simply Complex

**Figure 1:**
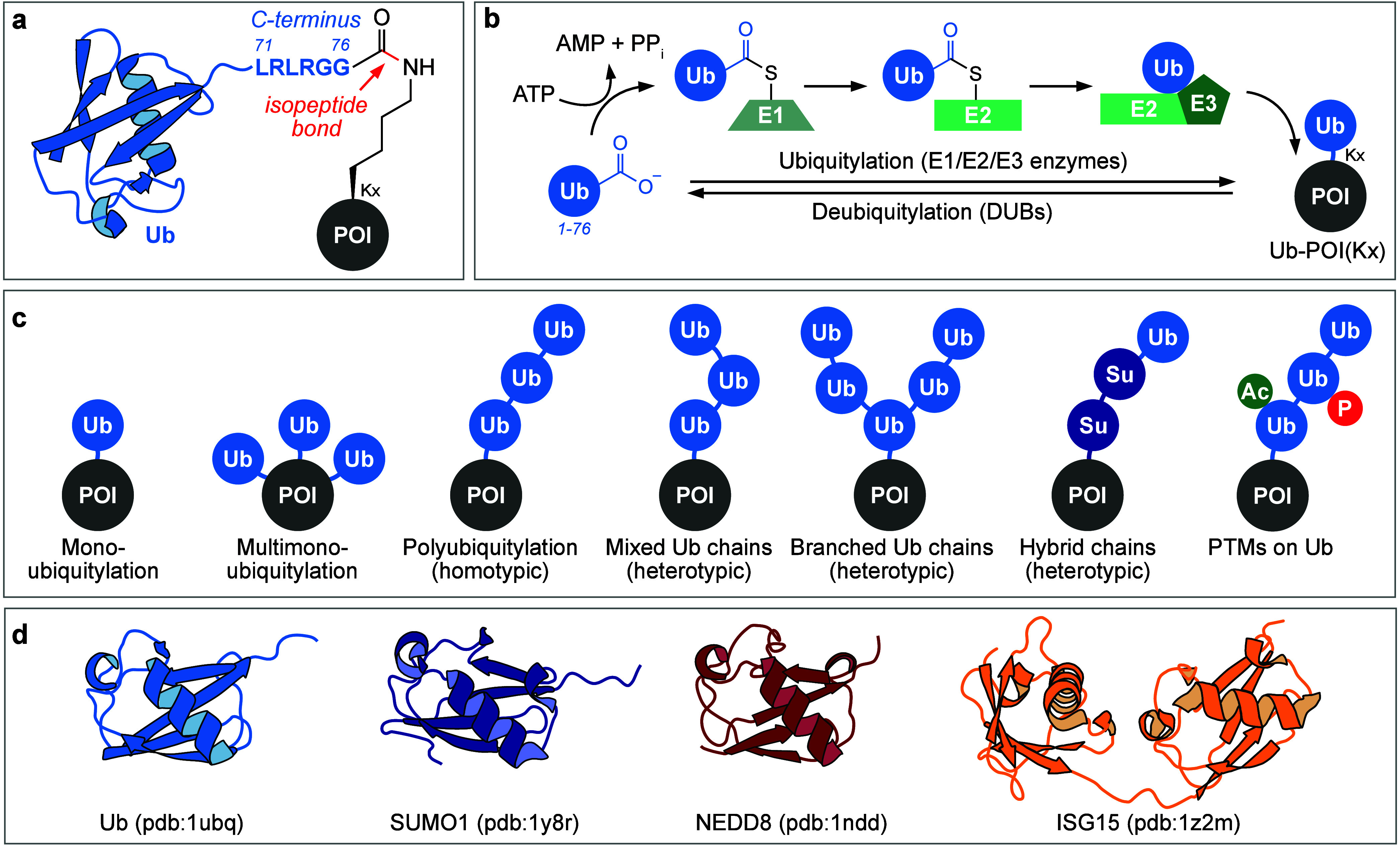
Ubiquitylation and different Ub topologies. (a) Schematic representation of a Ub-POI (protein of interest) conjugate. Ub is connected by its six-residue flexible C-terminus (LRLRGG) to a lysine residue of a POI via an isopeptide bond. (b) Enzymatic ubiquitylation/deubiquitylation. Ub gets charged onto the active site cysteine of an E1 enzyme in an ATP-dependent manner, followed by a transthioesterification onto an E2 enzyme. E3 ligases mediate the transfer of Ub onto a substrate protein. DUBs reverse the process by cleaving Ub from the target protein. (c) Different Ub/Ubl topologies. Mono- and multi-monoubiquitylation are characterized by the attachment of Ub-monomers. Polyubiquitylation events can be divided into homotypic (all Ubs are connected via the same type of linkage) and heterotypic architectures (chains with mixed types of linkages and branched chains). Hybrid chains involve other Ubls within the architecture. Modification of Ub topologies with small chemical PTMs such as acetylation and phosphorylation creates an additional layer of complexity. Ub is depicted as a sky blue circle, SUMO is depicted as a royal blue circle, acetylation is depicted as a small teal circle, and phosphorylation is depicted as a small red circle. (d) Cartoon representations of different Ubls with their characteristic β-grasp fold (also called Ub fold). Ub (PDB 1ubq) in sky blue, SUMO1 (PDB 1y8r) in royal blue, NEDD8 (PDB 1ndd) in red, and ISG15 (PDB 1z2m), which contains two Ub-like domains, in orange.

Proteins are the key players in all cellular life, and they perform a variety of essential functions from biocatalysis and signal transduction to scaffolding and transporting purposes. In order to balance and regulate these various processes, organisms adjust the abundance and status of their proteins in response to exogenous and endogenous stimuli. Proteomic diversity and flexibility are achieved both on a transcriptional level, for example, by changing protein expression patterns and/or through alternative splicing, and on a translational level, for example, through the installment of PTMs (post-translational modifications).^1,2^ Most PTMs involve the covalent modification of amino acid side chains of target proteins with small chemical functionalities such as acetyl, methyl, or phosphate groups. In contrast to these, larger and more complex PTMs like glycosylation and ubiquitylation also play important roles in regulating cellular processes.^3,4^ Ubiquitylation consists of the covalent attachment of the small protein Ub (ubiquitin) to target proteins and represents one of the most versatile and abundant PTMs in eukaryotes. Ub is a globular, 76 amino acid long protein and is nearly completely conserved across all eukaryotic species. In canonical ubiquitylation, Ub is attached to its substrate protein via an isopeptide bond formed between the C-terminal carboxylate of Ub and the ε-amino group of a lysine within the target protein (Figure 1a). Ubiquitylation controls a variety of cellular processes ranging from proteasomal degradation^5^ to DNA repair, endocytosis, autophagy, nuclear transport, and chromosomal organization.^6^ As ubiquitylation is closely intertwined with nearly all aspects of eukaryotic biology, its dysregulation can lead to numerous severe pathologies such as cancer or neurodegenerative disorders.^7^

The significance and complexity of ubiquitylation are demonstrated by the sophisticated machinery of enzymes that cells dedicate to the installation (writer proteins), interpretation (reader proteins), and reversal (eraser proteins) of Ub modifications. To achieve ubiquitylation, Ub is first activated, then conjugated, and finally ligated to target proteins by an enzymatic cascade comprised of E1 Ub-activating, E2 Ub-conjugating, and E3 Ub-ligating enzymes (Figure 1b). The human genome encodes two E1 enzymes, approximately 30 E2s, and an estimated number of more than 600 E3 ligases. In an ATP-dependent reaction, an E1 enzyme first generates a reactive Ub-AMP intermediate that is charged onto its active site cysteine forming an E1∼Ub thioester conjugate. One of the E2 enzymes then engages in a transthioesterification reaction to transfer Ub from E1 to the E2’s active site cysteine. The final transfer of Ub from the Ub∼E2 complex to the target protein is mediated by an E3 that recognizes a specific lysine residue in a specific substrate protein. Historically, E3s have been grouped into HECT (Homologous to the E6-AP Carboxyl Terminus) and RING (Really Interesting New Gene) type enzymes that catalyze Ub transfer through different mechanisms.^8,9^ While HECT-type E3 ligases form a thioester-linked Ub∼HECT intermediate, RING-type E3s mediate the direct transfer of Ub from the E2 enzyme to the target protein. The family of RBR (RING-between-RING) type E3 ligases catalyze ubiquitylation in a multistep mechanism that shares the characteristics of both HECT- and RING-mediated ligation. The RING1 domain recognizes the Ub∼E2 complex and transfers Ub to the active site cysteine located in the RING2 domain, which in turn catalyzes the transfer of Ub to the substrate protein.^10^ The recently discovered RCR (RING-Cys-relay) type E3 family, which so far contains MYCBP2 (MYC binding protein 2) as the sole member, exerts a unique mechanism involving two catalytic cysteines that relay Ub before its transfer onto the target protein. Remarkably, MYCBP2 was discovered to possess esterification activity, leading to noncanonical threonine and serine ubiquitylation within target proteins.^11^ RNF213 (ring finger protein 213) represents another exceptional type of E3 ligase that combines RING and AAA (ATPase associated with a variety of cellular activities) domains. Its ubiquitylation mechanism depends on a catalytic cysteine located in the zinc finger domain, and surprisingly, RNF213 was found to likely ubiquitylate the lipid A moiety of lipopolysaccharide during bacterial infection, constituting one of the first reported nonproteinaceous substrates to be ubiquitylated.^12−14^ While the long-standing consensus is that ubiquitylation is exclusive to lysine residues within target proteins, these recently discovered noncanonical ubiquitylation events and their novel cellular functions have attracted more and more attention as of late.^15−17^ Alluringly, noncanonical ubiquitylation is frequently installed by pathogenic effector proteins that interfere with the host Ub system to advance successful infection.^18−21^ The development of robust methods to detect and study these nonlysine and especially nonprotein ubiquitylation events is a major challenge in the emerging field but will represent a new milestone in understanding the complex Ub system.^22^

Ubiquitylation of a substrate protein may occur in the form of monoubiquitylation or multi-monoubiquitylation, where a single Ub monomer is attached to a single lysine residue or several lysine residues within the substrate protein, respectively (Figure 1c). As Ub can serve as a substrate for ubiquitylation itself, these modification patterns are expanded by polyubiquitylation: the modification of Ub with Ub chains. Ub displays eight potential ubiquitylation sites: the N-terminus and seven lysine residues (K6, K11, K27, K29, K33, K48, and K63) that can all participate in Ub chain formation. Ub chains linked via the same type of lysine residue are classified as homotypic chains. Conversely, heterotypic chains feature different linkage types and can be subdivided into mixed chains and branched chains, the latter featuring Ub monomers with more than one ubiquitylation site (Figure 1c).^23^

The resulting multitude of Ub topologies, also referred to as the Ub code,^4^ are deciphered by so-called reader proteins that use UBDs (Ub-binding domains) to interact with specific surface areas of Ub, *e.g.*, the hydrophobic F4, I36, and I44 patches or the T9/K11 TEK-box motif. UBDs of more than 20 different families, including UBAs (Ub-associated domains), UIMs (Ub-interacting motifs), and UBZs (Ub-binding zinc fingers), are found in many proteins.^6,24^ Typically, UBDs interact with monoUb with low affinities in the 10–500 μM range.^24^ This low affinity of a single UBD for monoUb allows for avidity effects in proteins that contain multiple UBDs, leading to fine-tuned affinity and selectivity toward distinct Ub topologies. While the well-studied K48-linked chains mediate proteasomal degradation,^25^ K63-linked chains have been found to regulate nondegradative functions exemplified by their important roles in signal transduction pathways.^26,27^ The remaining atypical linkages via M1, K6, K11, K27, K29, and K33 are less-well studied but have been linked to various signaling pathways in mitophagy, autophagy, epigenetics, and angiogenesis in recent years.^28^ Similarly, the biological functions of mixed and branched chains remain rather elusive and are still under investigation.^23,29,30^

In addition to the writers and readers of the Ub code discussed above, the Ub system also encompasses erasers: DUBs (deubiquitylating enzymes, Figure 1b) reverse ubiquitylation by hydrolyzing the isopeptide bond between Ub and the substrate protein, thereby allowing the tight regulation of ubiquitylation in response to internal and external stimuli. Human cells encode approximately 100 different DUBs that are classified into seven structurally different families: six cysteine protease families including UCHs (ubiquitin C-terminal hydrolases), USPs (ubiquitin-specific proteases), OTUs (ovarian tumor proteases), Josephins, MINDYs (motif interacting with ubiquitin (MIU)-containing novel DUB family), and ZUP1 (zinc finger containing ubiquitin peptidase 1), as well as one Zn-dependent metalloprotease family (JAMMs (JAB1/MPN/MOV34)).^31,32^ In addition to reverting ubiquitylation on target proteins, DUBs process precursor forms of Ub and contribute to maintaining Ub homeostasis by disassembling free polyUb chains.^32,33^ The specificities of DUBs toward different Ub topologies, their expression levels, and their distinct localization regulate fundamental cellular processes such as cellular propagation, DNA repair, and immune signaling.^34,35^ Further, it should be noted that besides their catalytic role, multiple DUBs of the OTU and USP families also possess diverse crucial noncatalytic moonlighting functions that are only starting to be investigated in more detail.^36^ Alterations and deregulation in DUB abundance and activity are therefore connected to various pathologies, and consequentially DUBs have become a focal point in drug discovery in recent years.^37,38^ This research is not limited to eukaryotic DUBs but also expands to bacterial and viral effector proteins that either exert DUB activity or modulate host DUBs to increase infectivity.^39−41^

The elegant concept of the Ub code is not limited to Ub itself: eukaryotes possess more than a dozen Ubls (Ub-like proteins), such as SUMO (small ubiquitin-related modifier), NEDD8 (neural-precursor-cell-expressed developmentally down-regulated 8), and ISG15 (interferon-stimulated gene 15 ubiquitin-like modifier), that share the ß-grasp fold and the flexible C-terminus of Ub (Figure 1d). Mirroring ubiquitylation, the activation and conjugation of Ubls to substrate proteins (ublylation) are concerted by Ubl-specific sets of E1, E2, and E3 enzymes and reversed by respective deconjugating enzymes.^42^ Ubls possess distinctive cellular functions but are also heavily intertwined with the Ub code, as ublylation can lead to crosstalk with ubiquitylation in a synergistic or antagonistic fashion. Moreover, the existence of polymeric hybrid chains (Figure 1c) containing different Ubls (*e.g.*, Ub and SUMO or Ub and NEDD8) represents an intriguing example of the overlapping worlds of Ub and Ubls.^43^ Another layer of complexity is conferred by small-molecule PTMs (Figure 1c).

As Ub and Ubls are protein-based PTMs, they can be targeted themselves by small-molecule modifications.^44^ It has, for example, been shown that at least six of the seven lysine residues within Ub are targets for site-specific acetylation, and serine/threonine and tyrosine residues of Ub are susceptible to phosphorylation.^23^

Considering the tremendous complexity of the Ub system, deciphering the entire Ub code strikes one as a Sisyphean mission. Nevertheless, chemists and biologists alike set out to unravel the mechanisms by which ubiquitylation dictates the fate of single proteins, cells, or entire organisms. Progress within this multidisciplinary adventure will not only reward us with a better understanding of eukaryotic life itself but also help us elucidate how dysfunctions of the Ub system are connected to disease and how pathogens hijack ubiquitylation patterns in host cells to increase virulence.

To gain further insights into how Ub and Ubls orchestrate cellular processes, we require a molecular and mechanistic understanding of how specific Ub/Ubl modifications affect target proteins and how different Ub/Ubl patterns are written, read, and decoded. For this it is important to create defined conjugates with Ub/Ubl attached to specific lysine residues within a protein of interest (POI). However, the generation of such defined and homogeneous Ub-POI conjugates poses several challenges, as the exact writers of specific ubiquitylation events are often unknown or the corresponding enzymes lose their specificities in *in vitro* settings. Progress in protein semisynthesis and chemical protein synthesis for the generation of various Ub-POI conjugates has paved the way for studying structural and mechanistic features of site-specific Ub attachments to proteins.^45−51^ The scope of these methods is, however, mostly limited to *in vitro* ubiquitylation of small and refoldable proteins that withstand harsh chemical deprotection protocols. Hence, there is a demand for innovative methodologies that on the one hand allow the generation of ever more complex Ub/Ubl conjugates, including complex Ub/Ubl chains attached to nonrefoldable multidomain substrate proteins, and on the other hand may even enable the generation of these specific conjugates within their native environment: inside of a eukaryotic cell. GCE (genetic code expansion) methodologies paired with bioorthogonal chemistries and/or enzymatic approaches may account for both of these challenges. GCE allows the modification of proteins in living cells by site-specifically introducing artificial designer amino acids into target proteins and therefore represents a promising tool to further shed light on Ub/Ubl signaling.

### 1.2 Expanding the Genetic Code: Naturally Unnatural

Proteins across all domains of life are built almost exclusively from the same set of 20 natural, also termed proteinogenic, amino acids. Pyl (pyrrolysine) and Sec (selenocysteine), sometimes referred to as the 21st and 22nd proteinogenic amino acids, represent rare exceptions. In spite of their substantial structural and functional role, proteinogenic amino acids cover only a limited range of chemical functionalities. Naturally they are complemented by PTMs and cofactors that significantly increase the chemical repertoire of proteins, enabling the diverse chemistry that defines life. To artificially increase the chemical space of proteins, scientists have strived for decades to develop approaches to endow proteins with unique chemical and biophysical probes in form of ncAAs (noncanonical amino acids). The use of solid-phase synthesis,^52^ native chemical ligation,^53^ and *in vitro* translation^54,55^ allows the site-specific installation of ncAAs into proteins. Such methods, however, are not only experimentally challenging and often low-yielding but also limited to the production of small proteins in *in vitro* settings. Strategies harnessing the translational machinery of a host organism to introduce ncAAs into proteins by GCE aim to overcome these boundaries. These approaches allow the residue- or site-specific incorporation of ncAAs into proteins during ribosomal translation.^56−60^

**Figure 2:**
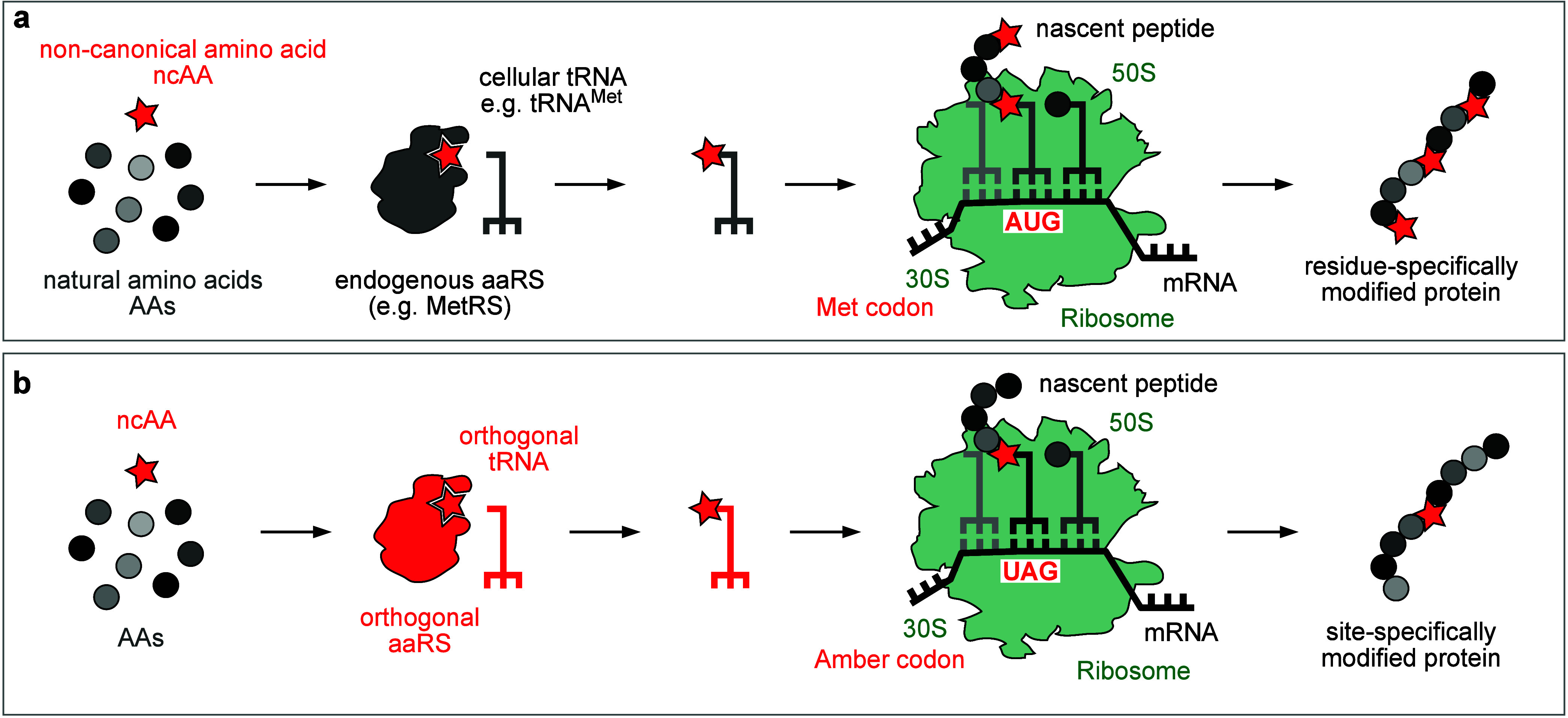
Incorporation of ncAAs (noncanonical amino acids) into proteins using the translation machinery. (a) Schematic representation of residue-specific incorporation of ncAAs into proteins. An isostructural ncAA is charged onto an endogenous tRNA by the enzymatic action of the corresponding endogenous aaRS, leading to globally and residue-specifically modified proteins. (b) Amber suppression makes use of an orthogonal aaRS that exclusively charges an ncAA onto an orthogonal tRNA bearing an amber anticodon. Ribosomal translation leads to incorporation of the ncAA in response to an amber stop codon introduced into the gene of interest, leading to a site-specifically modified protein. ncAA is represented by a red star, and natural amino acids are represented by gray circles.

Residue-specific incorporation of ncAAs via selective pressure allows global, proteome-wide decoration of proteins with specifically designed noncanonical functionalities. This approach relies on the recognition of an amino acid analogue as a substrate for an endogenous aaRS (aminoacyl-tRNA synthetase), leading to partial replacement of one of the 20 natural amino acids with an ncAA (Figure 2a). Typically, auxotrophic *Escherichia coli* (*E. coli*) strains, lacking the endogenous machinery for the biosynthesis of the natural amino acid that is to be replaced, are used, and POI expression is conducted in media depleted of the respective natural amino acid. The endogenous *E. coli* MetRS (methionyl-tRNA synthetase), for example, recognizes a range of different ncAAs, including Aha (l-azidohomoalanine), and has been used to globally incorporate Aha into proteins in response to the methionine sense codon (AUG).^61^ While the range of amino acid substrates that an endogenous aaRS can recognize is limited, it can be significantly expanded by introducing a mutant aaRS into the host organism,^62−70^ thereby creating proteins harboring more diverse functionalities.

Site-specific incorporation of ncAAs, on the other hand, relies on an orthogonal aaRS that specifically charges the desired ncAA but none of the 20 natural amino acids onto its cognate orthogonal tRNA and, consequently, directs its incorporation during mRNA translation in response to a blank codon placed at a defined site (Figure 2b). Most often, the amber stop codon (UAG) is the codon of choice for this approach—thus known as “amber suppression”—for multiple reasons: The amber codon terminates only 9% of *E. coli* genes, most of them being nonessential,^71,72^ and is only recognized by one of the two bacterial release factors that facilitate translation termination (RF1),^73^ which was also found to be nonessential in *E. coli*.^74^ Furthermore, certain species such as methanogenic archaea utilize the amber codon to introduce an amino acid instead of for terminating protein synthesis.^75−78^ To be classified as orthogonal, an aaRS/tRNA pair imported from a heterologous host must not interfere with endogenous aaRS/tRNA pairs and amino acids. Hence, the orthogonal aaRS selectively and exclusively charges the ncAA onto the orthogonal tRNA, and the orthogonal tRNA is not aminoacylated by any endogenous aaRS.

Over the last two decades, a wide variety of different aaRS/tRNA pairs have been discovered and engineered for the site-specific incorporation of ncAAs into proteins. These aaRS/tRNA pairs typically originate from evolutionarily distant organisms with respect to the host organism of choice. Among the most prevalently used orthogonal aaRS/tRNA pairs are the PylRS/tRNA (pyrrolysyl-aaRS/tRNA) pairs derived from the archaeal species *Methanosarcina mazei* (*Mm*) and *Methanosarcina barkeri* (*Mb*), which were shown to retain orthogonality and specificity in pro- and eukaryotic expression systems.^58−60,79−83^ Next to *Mm*- and *Mb*PylRS/tRNA pairs, variants of the *Methanocaldococcus jannaschii* (*Mj*) TyrRS/tRNA (tyrosyl-aaRS/tRNA) synthetase pair, which is orthogonal in *E. coli* and other prokaryotes but not eukaryotic systems, have found wide applications for incorporating ncAAs in *E. coli*(58,84) and constitute the first orthogonal aaRS/tRNA pair for the incorporation of ncAAs in living *E. coli*.^76^ Analogously, the pSRS (phosphoseryl-aaRS) from *Methanococcus marplaudis* (*Mmp*) has been combined with the cysteinyl-tRNA from *M. jannaschii* to engineer an orthogonal aaRS/tRNA pair for the site-specific incorporation of pS (phosphoserine), its nonhydrolyzable analogues, and pT (phosphothreonine) into proteins in *E. coli*.^85−88^ Vice versa, the *E. coli* leucyl-,^89^ tyrosyl-,^90^ and tryptophanyl-aaRS/tRNA pairs,^91^ which are not orthogonal in prokaryotes, were utilized to incorporate a broad spectrum of ncAAs in yeast and mammalian cells. Recent studies have furthermore designed chimeric orthogonal aaRS/tRNA pairs by grafting the N-terminal tRNA-binding domain of a PylRS variant onto the catalytic domain of the *E. coli* histidyl-, phenylalanyl-, and alanyl-aaRS in combination with transplanting the pyrrolysine tRNA acceptor stem to their respective tRNA counterparts. These chimeric aaRS/tRNA pairs can direct the site-specific incorporation of phenylalanine and tryptophane derivatives into proteins expressed in *E. coli*.^92−95^ The discovery of a PylRS/tRNA variant from *Methanomethylophilus alvus* (*Ma*) showed that some PylRS variants contain solely the C-terminal catalytic domain and are functional without the N-terminal tRNA binding domain (ΔNPylRS/tRNA). The *Ma*PylRS/tRNA pair was demonstrated to be active and orthogonal in *E. coli* and mammalian cells and to efficiently incorporate ncAAs.^96−98^ To enable site-specific incorporation of more than one distinct ncAA into a target protein, mutually orthogonal aaRS/tRNA pairs have been evolved either by taking advantage of the evolutionary divergence of the used aaRS/tRNA pairs or by creating mutual orthogonality via directed evolution.^99−102^ These orthogonal pairs have been leveraged for the suppression of two or even all three stop codons introduced into a POI.^99,103−105^ Recent efforts to even further expand the repertoire of available codons for ncAA incorporation include the evolution of an orthogonal bacterial ribosome that facilitates the suppression and translation of quadruplet codons^100,106,107^ and genome engineering efforts in *E. coli* to recode and repurpose sense codons for ncAA incorporation. Early studies focused on replacing all genomic amber codons in *E. coli* with synonymous ochre codons, allowing for the deletion of RF1 and the efficient reassignment of the amber translation function.^108^ Further engineering efforts, led by the Chin group, culminated in the recent creation of the synthetic *E. coli* strain syn61 in which two serine codons are substituted with their synonyms and the amber codon is replaced with the ochre codon.^109^ Removing the respective decoding tRNAs and RF1 from syn61’s genome created three blank codons, enabling the incorporation of three distinct ncAAs using mutually orthogonal aaRS/tRNA pairs.^110^ Most importantly, the substrate scope of PylRS and other orthogonal aaRS has been significantly expanded by directed evolution approaches to identify aaRS variants with high selectivity and specificity for a given ncAA. While the most frequently used evolution approaches rely on two-step dead or alive selection systems,^58,59,111−113^ recent developments showed that it is possible to directly evolve aaRS variants through phage-assisted continuous or noncontinuous evolution^114,115^ and tRNA display. The latter system that decouples aaRS evolution from translation was used to identify PylRS variants that direct the incorporation of α,α-disubstituted and β-amino acids, showing that the substrate scope of PylRS can be extended beyond l-amino acids with different side chains.^116^ Using orthogonal aaRS/tRNA pairs and their engineered variants, hitherto more than 460 ncAAs^59,117,118^ have been site-specifically incorporated into proteins in prokaryotic^58,59^ and/or eukaryotic cells,^81,83,90,117,119^ including multicellular organisms such as plants,^120^*Caenorhabditis elegans*,^121−123^*Drosophila melanogaster*,^124^ and *Mus musculus*.^125−129^

The ability to introduce novel functionalities site-specifically into POIs in living cells has shown great relevance for different fields as it allows the study and manipulation of biological processes that are difficult to tackle via more traditional approaches with impressive spatiotemporal control. The rapid rise of the reported applications of GCE in multiple research areas over the past decade is therefore not surprising and has been the topic of several reviews.^59,60,102,130−132^ To highlight only a couple of examples, GCE has proven to be an excellent means of site-specific protein labeling in both *in vitro* and *in cellulo* or *in vivo* settings. The approach consists of the incorporation of ncAAs equipped with a bioorthogonal handle (*e.g.*, alkynes, strained alkenes and alkynes, and azides or tetrazines, among others) that participates in a rapid and selective bioorthogonal reaction with an externally added small molecule that introduces a biochemical or biophysical reporter of choice.^60^ The use of light as an elegant external trigger for manipulating enzyme activity has also been explored within the scope of GCE. Several signaling processes in the cell have been successfully enlightened through the incorporation of photocaged^83,133,134^ or photoswitchable ncAAs.^135,136^ Photocrosslinking ncAAs have, on the other hand, allowed the mapping of weak and transient PPIs (protein–protein interactions).^137,138^ Similarly, ncAAs bearing fine-tuned electrophilic moieties that only react with nucleophiles in sufficient proximity have been employed for proximity-triggered chemical crosslinking to stabilize low-affinity PPIs.^138−141^ Furthermore, site-specific incorporation of ncAAs has found application in improving enzyme activity and stability, uncovering enzyme mechanisms, and designing enzymes with new catalytic functions and mechanisms.^142−146^

Notably, GCE can also be used to study the effects of PTMs through the direct incorporation of an ncAA resembling a specific PTM or a mimic thereof. This decouples the installation of a certain site-specific PTM from its cognate endogenous writer enzyme and enables the facile production of homogeneously modified proteins. It is crucial in case the writer enzymes are unknown, challenging to handle, or nonexistent, as some PTMs are installed in a nonenzymatic manner. In contrast to synthetic or semisynthetic approaches, GCE can be used to introduce PTMs on large, nonrefoldable proteins and on proteins in living cells, potentially enabling not only the introduction but also the investigation of these PTMs and their effects in native environments. GCE has allowed the site-specific incorporation of a variety of small-molecule PTMs,^147,148^ including acylations,^79,149−152^ methylation,^153−155^ phosphorylation,^85,87,156,157^ and sulfation,^158^ and has recently been the focus of an extensive review.^148^ In contrast to these small chemical PTMs, where corresponding ncAAs bearing these modifications or mimics thereof are suitable substrates for engineered orthogonal aaRS/tRNA pairs, protein-based PTMs like ubiquitylation and ublylation cannot be directly installed via cotranslational incorporation. Instead, ncAAs, most typically based on lysine derivatives, bearing a chemoselective reactive moiety for the attachment of Ub/Ubl have been designed and encoded via GCE. In combination with bioorthogonal conjugation and/or chemoenzymatic ligation techniques, these strategies afford access to site-specifically ubiquitylated POIs.

## 2 Exploiting GCE Approaches for Building Specific Ub-POI Conjugates

In this Review, we provide an extensive overview of how GCE can be leveraged to study the intricacies of the highly complex Ub/Ubl code. We present available GCE tools for generating Ub-POI conjugates and discuss their advantages, disadvantages, and applications. We hope that our synopsis may serve as a guideline and go-to reference for structural biologists, biochemists, chemical biologists, and cell biologists working in the Ub field, complementing other recent reviews in the field.^48,159,160^ We provide an overview of GCE methods for generating Ub-POI conjugates by dividing them into two groups, *i.e.*, (1) methods that generate Ub-POI conjugates with artificial linkages instead of the endogenous isopeptide bond and (2) methods resulting in isopeptide-linked conjugates, and discuss how the respective conjugates have been applied. Additionally, we describe how Ub and Ubls themselves are modified by small chemical PTMs and how GCE approaches may help the study of this second layer of PTMs. Furthermore, we discuss GCE-based tools tailored to the investigation of writers, interactors, and erasers of the Ub code. Finally, we summarize how GCE may help to address future challenges the Ub community has to face.

### 2.1 Strategies Resulting in Ub-POI Conjugates with Artificial Linkages

Bioorthogonal reactions have been used to generate ubiquitylated target proteins displaying an artificial linkage between Ub and POI instead of the endogenous isopeptide bond catalyzed by E3 ligases. By definition, bioorthogonal reactions must fulfill several essential requirements: (i) they must show high chemoselectivity of the reaction partners toward various chemical functionalities found on biomolecules and ideally within living cells, (ii) they should generate the reaction product in high yields and with fast kinetics under physiological conditions (aqueous environment, ambient temperature, and neutral pH), and (iii) the reaction partners have to be metabolically, kinetically, and thermodynamically stable.^161−163^ For generating Ub-POI conjugates, the product of the respective bioorthogonal reaction should further closely mimic the native isopeptide bond and be obtained ideally in quantitative yields within short incubation times in aqueous buffers on folded proteins without requiring too high a stochiometric excess of one of the reaction partners (Ub/Ubl or POI). Furthermore, the bioorthogonal functionalities have to be efficiently installed at the C-terminus of Ub/Ubl and incorporated within the POI at the chosen ubiquitylation/ublylation site. With respect to these criteria, strategies based on click reactions between alkynes and azides (section 2.1.1), oxime ligation reactions between aminooxy moieties and aldehydes or ketones (section 2.1.2), and thiazolidine ligation (section 2.1.3) have been developed to generate Ub-POI conjugates with artificial linkages and will be discussed in more detail below.

#### 2.1.1 Click Chemistry-Based Approaches

**Figure 3:**
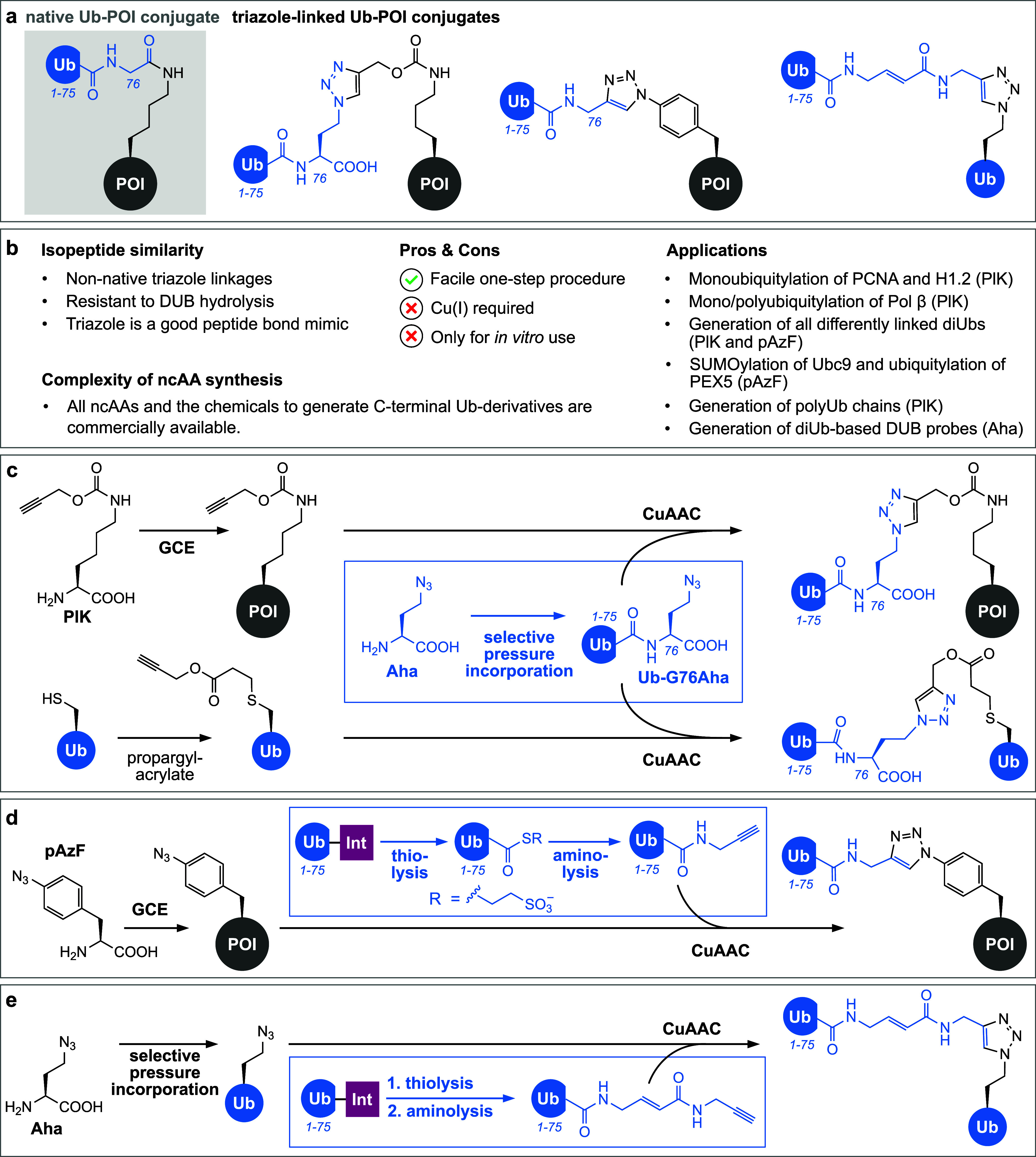
Click chemistry-mediated ubiquitylation. (a) Schematic representation of click chemistry-generated Ub-POI conjugates displaying triazole linkages instead of the native isopeptide bond as accessed via methodologies shown in subpanels c (left), d (middle), and e (right). (b) Summary of important characteristics and advantages/disadvantages of the presented click chemistry-based methodologies. (c) Combination of amber suppression and selective pressure incorporation. Site-specific installation of the alkyne moiety is achieved by incorporating PlK into a POI using amber suppression. Alternatively, the alkyne can be introduced via Michael addition of propargyl acrylate to a cysteine side chain. The complementary azide moiety is introduced into Ub at position G76 by selective pressure incorporation of Aha. CuAAC between the alkyne and the azide moiety results in a triazole-linked Ub-POI conjugate. (d) Combination of amber suppression and intein technology. Site-specific incorporation of pAzF into a POI using amber suppression is used to install the azide moiety. Thiolysis of an Ub(1–75)-intein fusion using sodium 2-mercaptoethanesulfonate (Mesna) yields a thioester Ub-SR that is subsequently used for aminolysis with propargylamine, resulting in Ub bearing an alkyne at its C-terminus. Subsequent CuAAC yields a triazole-linked Ub-POI conjugate. (e) Combination of selective pressure incorporation and intein technology allows the generation of diUb DUB probes. In order to introduce an azide moiety, Aha is incorporated into an acceptor Ub using selective pressure incorporation. In parallel, intein-derived Ub(1–75)-SR is treated with a Michael acceptor bearing a propargylamine derivative for aminolysis, resulting in Ub that bears both an electrophilic trap and a bioconjugation handle at its C-terminus. Subsequent CuAAC results in the formation of a triazole-linked diUb probe bearing a Michael acceptor at the linkage site.

The development of click chemistry and bioorthogonal chemistry, awarded with the Nobel Prize in Chemistry in 2022, introduced the versatile and powerful click reaction concept to chemical biology.^164,165^ The Cu(I)-catalyzed 1,3-dipolar cycloaddition between a terminal alkyne and an azide (CuAAC) affords 1,4-disubstituted 1,2,3-triazole-linked products and proceeds with fast kinetics in various complex chemical and biological settings. Leveraging GCE for the site-specific incorporation of correspondingly modified ncAAs enables the modification of recombinant proteins with azide or terminal alkyne moieties on recombinant proteins^80,166,167^ and has been utilized in a plethora of applications to label POIs with correspondingly modified biophysical probes such as fluorophores or affinity handles.^60,168^ As both azide and alkyne reaction partners can be incorporated into proteins, CuAAC can also be leveraged to generate protein–protein conjugates. Accordingly, the technology has been applied to generate various Ub-POI conjugates over the past decade following one of three general strategies (Figure 3a and b).

The first approach for generating defined homogeneous Ub-POI conjugates via CuAAC was developed by Marx, Rubini, and co-workers and relies on both selective pressure incorporation and amber suppression to introduce azide and alkyne moieties into Ub/Ubl and POI, respectively.^169^ Selective pressure incorporation utilizing the MetRS in *E. coli* enables the substitution of the C-terminal glycine (G76) of the donor Ub with the azide-bearing methionine analogue Aha (azidohomoalanine, Figure 3c).^61^ Site-specific incorporation of PlK (*N*ε-(propargyloxycarbonyl)-l-lysine, Figure 3c) affords the introduction of a terminal alkyne moiety into an acceptor POI.^169^ To access all seven triazole-linked diUbs (diubiquitins), PlK was individually incorporated at all seven lysine sites within an acceptor Ub using the wt (wild type) *Mb*PylRS/tRNA pair and reacted via CuAAC with the Aha-modified donor Ub(G76Aha).

Autoubiquitylation assays with the ubiquitin ligase E6-AP showed that diUbs generated with this method can engage in polyUb chain formation,^169^ confirming their structural similarity to isopeptide-linked diUbs. CuAAC between Aha and PlK was, however, not limited to diUb formation, as demonstrated by the generation of monoubiquitylated PCNA (proliferating cell nuclear antigen).^170^ During DNA replication, the ring-shaped homotrimeric PCNA encircles the DNA and tethers polymerases, *e.g.*, the high-fidelity Polδ (DNA polymerase-δ), to it, thereby enhancing polymerase processivity. Furthermore, PCNA plays an important role in mediating tolerance to DNA damage. In brief, DNA lesions cause stalling of the replication fork, which leads to monoubiquitylation of PCNA at K164 and thus to the recruitment of low-fidelity DNA polymerases (*e.g.*, Polη) that promote translesion synthesis. Notably, PCNA can also be modified differently, *e.g.*, by K63-linked Ub chains, SUMOylation, or decoration with ISG15, leading to different downstream responses.^171^ Incorporation of PlK at position K164 of PCNA followed by CuAAC with donor Ub(G76Aha) afforded triazole-linked Ub-PCNA(K164) that was shown to stimulate DNA synthesis by Polδ and to possess increased affinity for Polη, thus confirming the suitability of CuAAC-generated Ub-POI conjugates for biochemical research.^170^ Further optimization of the click reaction itself by adding nondenaturing amounts of anionic surfactants, such as SDS (sodium dodecyl sulfate), facilitated the site-specific ubiquitylation of Polβ (DNA polymerase-β) at K61.^172,173^ Intriguingly, it was also possible to generate bifunctional Ubs by replacing one lysine residue with PlK via amber suppression while also installing C-terminal Aha via selective pressure incorporation. These bifunctional building blocks were used to generate polyubiquitylated Polβ and to generate and study unanchored K11-, K27-, and K29-linked Ub chains.^174,175^ Incubation of these triazole-linked Ub chains with *Xenopus laevis* egg extracts showed their resistance toward DUB-catalyzed isopeptide hydrolysis and enabled studies on linkage-specific physiological effects of Ub chains. Ca^2+^-induced cyclin B degradation in *Xenopus laevis* egg extracts was inhibited by K11-linked chains, while K27- and K29-linked chains had no effect on cyclin B levels, and it was hypothesized that the binding of K11-linked Ub chains to the proteasome outcompeted the recruitment and degradation of natural substrates of the proteasome.^174^ In a different application, PlK was incorporated into histone H1.2 to generate site-specifically ubiquitylated histone H1.2.^176^ Histone H1.2 is one of the most prevalent human subtypes of linker histone H1 and is known to be heavily post-translationally modified. Ubiquitylation of H1 has been linked to activation of gene expression, DNA damage response, and antiviral protection. Methods to study this histone PTM in a controlled setting are therefore of high interest. PlK was incorporated into N-terminally tagged H1.2 at lysine position K17, K64, or K206, and CuAAC with Ub(G76Aha) afforded the ubiquitylated histone variants. Assessment of H1.2 interactomes retrieved from HEK293T lysates showed that around a fifth of the enriched interactors were ubiquitylation-dependent, including several known DUBs that were characterized in more detail in downstream biochemical assays. Furthermore, it was shown that ubiquitylation of H1.2 at K64 influences its interaction with SIRT1 (sirtuin 1), ultimately also affecting SIRT1 deacetylation activity *in vitro*. This observation in addition to the finding that Ub-H1.2(K64) seemed to promote a relaxed conformational chromatosome state led to the conclusion that site-specific ubiquitylation of histone H1 likely contributes to a transcriptionally active state, in line with previous reports.^176^ Research on H1.2 and modified H1.2 was expanded in a recent follow-up study that for the first time used intact chromatosomes as bait in affinity enrichment mass spectrometry interactome studies in HEK293T lysates.^177^

Recently, access to triazole-linked Ub chains was further simplified by installing the alkyne functionality onto Ub via chemical modification of cysteines instead of site-specific incorporation of PlK.^178^ The acceptor lysine of Ub was mutated to cysteine, followed by Michael addition with propargyl acrylate and subsequent CuAAC with Ub(G76Aha) (Figure 3c).

This strategy facilitated the preparation of all seven differently linked DUB-resistant polyUb chains, which were used for the identification of novel interactors of K27-, K29-, and K33-linked chains through affinity-based proteomic profiling.^178^ NMR studies of triazole-linked K48-diUb demonstrated the close structural resemblance to the natively linked K48-diUb, confirming click chemistry approaches as attractive tools for identifying native Ub chain interactors.^179^ Similarly, K11-linked and K27-linked diUbs, as well as K11-linked Ub trimers, were generated with triazole linkages and investigated in regard to their structural and functional characteristics using NMR spectroscopy and MD simulations.^180,181^ In another recent study, the Scheffner and Marx groups showed that multiple lysine-to-cysteine replacements in Ub followed by reaction with propargyl acrylate and CuAAC with Aha-modified Ub allowed the generation of Ub chains with defined branching sites at K6/K11, K11/K48, K11/K63, and K6/K11/K48 residues.^182^ As expected, the triazole-linked branched Ub oligomers were resistant toward DUB-catalyzed hydrolysis in eukaryotic cell lysates, potentially making them ideal probes for identifying proteins that selectively interact with defined branched Ub topologies in cell lysates. Taken together, the cysteine mutagenesis strategy is technically less demanding and higher yielding than the site-specific incorporation of PlK by amber suppression. It is, however, limited to the ubiquitylation of target proteins that do not contain endogenous cysteine residues and has so far only been applied for building diverse Ub topologies and not for the ubiquitylation of other target proteins.

The second main strategy for generating Ub- and Ubl-POI conjugates with GCE is based on a combination of amber suppression and intein technology (Figure 3d). As mentioned above, one gene can encode multiple proteoforms that differ in sequence length, which can, for example, be caused by alternative RNA splicing.^1^ Splicing can, however, also occur post-translationally on the protein level as first observed for the yeast *TFP1* gene product.^183,184^ Protein splicing is autocatalyzed by protein domains called inteins that excise themselves from precursor proteins upon ligation of the flanking N- and C-terminal sequences called N- and C-exteins. The exact mechanism of protein splicing varies among distinct intein classes but generally involves a series of acyl shifts and *trans*-(thio)esterifications.^185−187^ The discovery that that introduction of specific point mutations into the intein sequence stalls protein splicing in a (thio)ester state^188^ opened up the possibility to obtain POIs with C-terminal thioesters by replacing the N-extein with a POI. Addition of a small molecule containing a nucleophilic thiol group leads to *trans*-thioesterfication and thereby affords the POI as the C-terminal thioester of the small molecule.^189^ Such C-terminal thioesters are integral for semisynthesis of proteins via EPL (expressed protein ligation),^190^ but they can also undergo aminolysis with suitable small molecules containing a nucleophilic amine group.^186^ This allows the introduction of various functionalities to the Ub C-terminus,^191^ including electrophilic warheads used for DUB trapping^192^ or terminal alkynes suitable for CuAAC. The Mootz group used an intein-generated C-terminal thioester of a SUMO2 variant lacking the last two glycine residues (SUMO2(ΔGG)) to install a terminal alkyne on the Ubl via aminolysis with propargylamine and demonstrated successful CuAAC ligation to a small target protein that was previously chemically equipped with an azide moiety through cysteine-reactive chemistry.^193^ In several follow-up studies by the same group, the established intein technology was used to install a C-terminal alkyne on Ubl variants lacking the C-terminal G (Ubl(ΔG)) by incubation of the respective Ubl-thioester with propargylamine. In contrast to previous approaches, the azide moiety was introduced into the acceptor POI via GCE using the ncAA pAzF (*p*-azido-l-phenylalanine, Figure 3d) and the previously evolved mutant *Mj*TyrRS/tRNA pair,^167^ broadening the scope of this approach to substrate proteins containing cysteine residues. This technology was used to generate a triazole-linked conjugate between SUMO2 and the SUMO-conjugating enzyme Ubc9 (UBE2I) by incorporating pAzF into Ubc9, followed by CuAAC with a C-terminally alkyne-modified SUMO2(ΔG) variant. SUMOylation assays with substrates Sp100 (nuclear autoantigen Sp-100) and RanGAP (Ran GTPase-activating protein) indicated altered the substrate preference of SUMOylated Ubc9 versus its unmodified counterpart in accordance with biochemical data on endogenously SUMOylated Ubc9.^194^ Furthermore, all seven triazole-linked diUbs were generated with this method in a facile manner, although yields for diUb formation did not exceed 20–40%. As expected, the triazole-linked diUbs proved to be stable against DUB cleavage but importantly showed no decreased binding toward the UBA domain of Mud1, a DNA damage response protein that specifically recognizes K48-linked chains through its UBA domain.^195^ The structural and dynamic integrity of a triazole-linked diUb, accessed via the pAzF-based approach, was further confirmed by a study based on quantum mechanical and molecular mechanical methods.^196^ In another recent study, pAzF and intein chemistry were used to generate a triazole-linked conjugate between Ub and PEX5 (peroxisomal targeting signal 1 receptor).^197^ Peroxisome biogenesis and turnover rely on the import of peroxisomal matrix proteins into peroxisomes, which is mediated by the import receptors PEX5 and PEX7. Upon cargo release, PEX5 is monoubiquitylated at C11, forming a thioester-linked Ub-PEX5(C11) conjugate and leading to PEX extraction from the peroxisomal membrane followed by cytosolic deubiquitylation to initiate the next transport cycle. Studies in ΔPEX5 fibroblasts electroporated with the *in vitro* generated triazole-linked Ub-PEX5(C11) conjugate revealed that the artificially linked Ub-PEX5(C11) conjugate was able to bind to the PEX7 complexes and restore cargo import, mimicking the behavior of natively linked Ub-PEX5(C11).^197^

A third click chemistry-based approach introduced by Kessler, Kramer, and co-workers combines the features of the two presented technologies to decorate Ub and Ubls with azide and alkyne moieties. It relies on site-specific incorporation of Aha at acceptor lysine sites within Ub, using selective pressure incorporation via MetRS, and intein-aided installation of a terminal alkyne moiety bearing an electrophilic warhead at the C-terminus of Ub (Figure 3e).^198^ The CuAAC-generated triazole-linked diUbs possess an acrylamide moiety in their linker region and can act as DUB inhibitors by covalently trapping the active site cysteine of DUBs, which renders them useful probes for activity-based DUB profiling in eukaryotic cell lysates. Surface plasmon resonance experiments of these activity-based diUb probes with an active site mutant of the DUB OTUB1 confirmed similar binding behavior as seen for their respective isopeptide-linked counterparts, proving the utility of these artificially linked conjugates for DUB profiling. Treatment of HEK239T cell lysates with the acrylamide-bearing diUb probes followed by pull-down and mass spectrometry analyses revealed that many DUBs displayed a preference for atypical linkages (M1, K11, K27, and K29 linkages) over the well-studied K48 and K63 linkages.^198^

Taken together, click chemistry-based approaches for studying protein ubiquitylation have been optimized extensively over the past years and have proven to be well-suited technologies for generating and studying Ub-POI conjugates. All strategies discussed above yield nonhydrolyzable Ub-POI conjugates. This enables their application in complex environments, *e.g.*, cellular extracts containing endogenous DUBs that would process natively linked Ub-POI conjugates. Despite the different linker lengths between POI (or acceptor Ub) and donor Ub/Ubl in the various CuAAC-generated Ub/Ubl-conjugates, their similarity and resemblance to natively linked conjugates were confirmed *in silico* and/or experimentally for various Ub/Ubl-POI conjugates. The CuAAC approaches based on intein technologies can in principle also be extended to other Ubls, while selective pressure incorporation of Aha is limited to Ubls that do not contain internal methionine residues and can therefore not be expanded to, *e.g.*, SUMO, ISG15, and NEDD8. A similar constraint holds true for the approach that introduces the alkyne moiety into target proteins via the reaction of cysteine with propargyl acrylate: it is limited to substrate proteins that do not contain any cysteine residues or in which cysteine residues can be mutated without perturbing the structure and function of the protein.^178^ In addition, CuAAC relies on cytotoxic Cu(I), which limits the method to *in vitro* applications and can also affect the integrity of purified proteins, as metal traces may lead to oxidation or precipitation.^199^ Nevertheless, the strength of click chemistry-based approaches lies in their ease-of-use and simplicity, as all components are commercially available and the procedures can be implemented easily in a typical biochemistry/structural biology lab to access Ub/Ubl-POI conjugates.

#### 2.1.2 Oxime Ligation-Based Approaches

Condensation reactions between aldehydes or ketones and primary amine nucleophiles yielding imines, hydrazones, or oximes have found broad application as bioconjugation methods on peptides and purified proteins.^200^ The oxime ligation between carbonyls and aminooxy groups is of particular interest, as the obtained oxime bond is less prone to hydrolysis compared to imines and hydrazones.^201^

**Figure 4:**
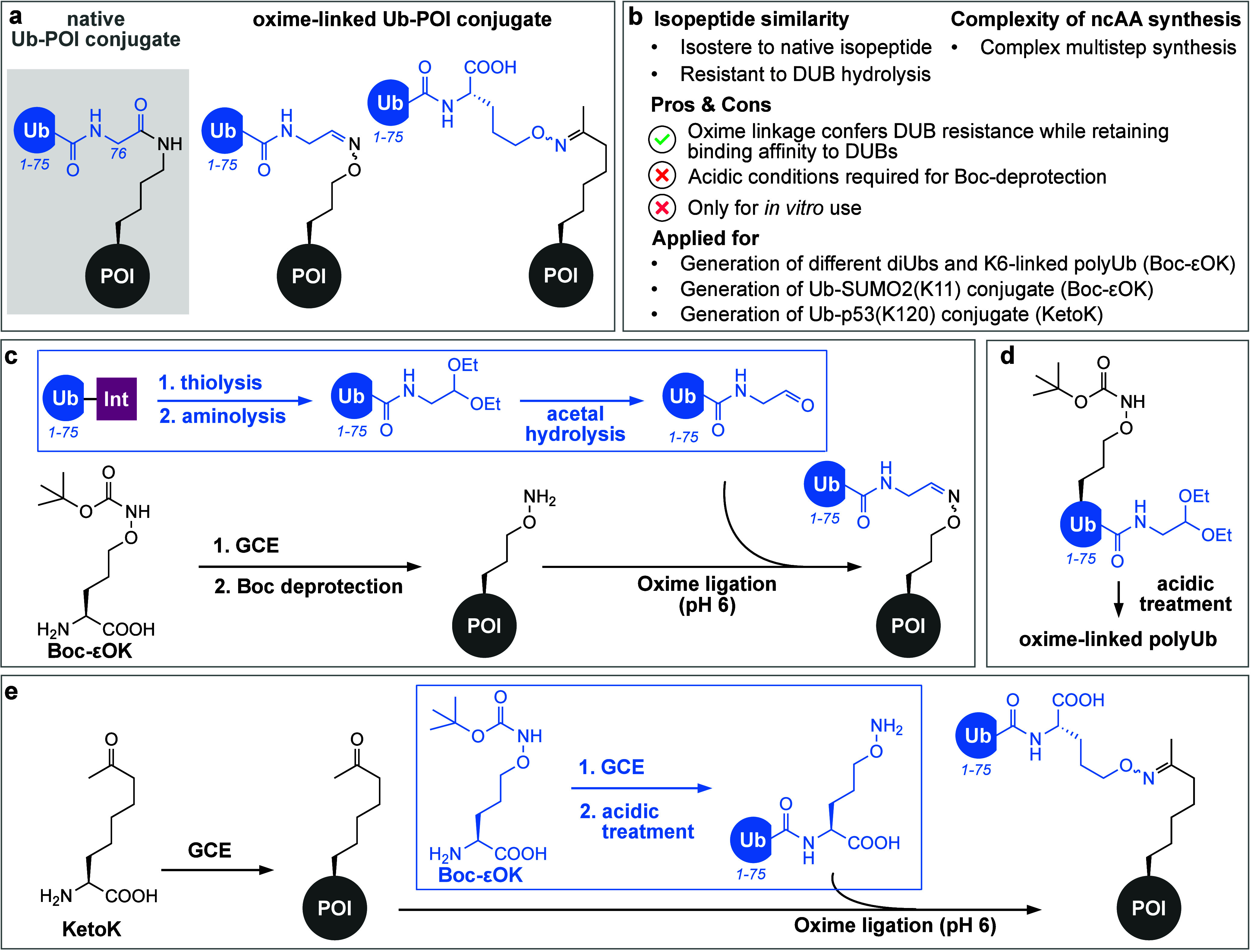
Oxime ligation-mediated ubiquitylation. (a) Schematic representation of Ub-POI conjugates displaying an oxime linkage. (b) Summary of important characteristics, advantages, disadvantages, and applications of oxime ligation-mediated ubiquitylation. (c) Intein technology enables access to Ub(1–75)-thioester, which can be converted to a Ub(1–75)-acetal by aminolysis. Subsequent acidic acetal hydrolysis yields Ub bearing a C-terminal aldehyde moiety. In parallel, amber suppression facilitates the site-specific incorporation of Boc-εOK into a POI. TFA treatment of the Boc-εOK-bearing POI is used to remove the Boc protection group, revealing the reactive aminooxy moiety. Oxime ligation between the aminooxy-bearing POI and Ub with a C-terminal aldehyde moiety at slightly acidic pH results in an oxime-linked Ub-POI conjugate. (d) Access to polyUb chains is possible by introducing both functionalities, the Boc-protected aminoxy moiety and the C-terminal acetal, into one Ub molecule. Acidic treatment leads to Boc deprotection and acetal hydrolysis, which allows polymerization via oxime ligation. (e) Site-specific incorporation of the ncAA KetoK represents the possibility to install a ketone in a nonrefoldable POI that can undergo oxime ligation with an aminooxy-bearing Ub.

In the context of generating Ub/Ubl-POI conjugates, the oxime ligation confers an added advantage, as the oxime linkage presents a close mimic of a peptide bond but provides stability against DUBs (Figure 4a and 4b). Initial efforts to create oxime-linked Ub-POI conjugates focused on ligating a Ub variant, which lacked the two C-terminal glycine residues (UbΔGG) and was modified with a C-terminal aldehyde functionality, to aminooxy-bearing Ub-fragment peptides generated via SPPS (solid phase peptide synthesis).^202^ Virdee and co-workers then expanded the approach to expressed proteins by site-specifically installing an aminooxy moiety in a target protein via amber suppression.^203^ Incorporation of Boc-εOK (*N*ε-(*tert*-butyloxycarbonyl)aminooxy-l-lysine, Figure 4c) was achieved by using a *Mb*PylRS/tRNA variant.^204^ The Boc (*tert*-butoxycarbonyl) protecting group of Boc-εOK provides a recognition element for the orthogonal synthetase and prevents side-reactions and adduct formations with cellular ketones such as pyruvate. Boc deprotection under acidic conditions on the purified protein unmasks the aminooxy moiety in the acceptor POI that can subsequently undergo oxime ligation with an appropriately C-terminally modified Ub/Ubl variant.^203^ Functionalization of the donor Ub C-terminus was achieved through aminolysis of an intein-derived Ub-thioester with an acetal-bearing small molecule that was *in situ* converted to the respective C-terminal aldehyde under acidic conditions.^191^

The strategy was successfully employed for the generation of oxime-linked K6- and K48-diUbs, as well as a Ub-SUMO2(K11) conjugate. Comparison of X-ray crystal structures of isopeptide- and oxime-linked K6-diUbs confirmed that the oxime linkage provides a suitable mimic for the isopeptide linkage. The oxime linkage was only observed as a *trans* regioisomer, accurately mimicking the native isopeptide conformation. Indeed, the oxime-linked conjugates showed high binding affinities toward tested DUBs but remained refractory to DUB-catalyzed hydrolysis, rendering them promising DUB inhibitors.^203^ IC_50_ measurements confirmed oxime-linked K6-diUb, Ub-SUMO(K11), and K48-diUb were potent inhibitors for the promiscuous DUB UCHL3. Furthermore, nanomolar affinities between oxime-linked K48-diUb and the DUB USP2 were determined, in agreement with affinities measured for natively linked K48-diUb.

Inspired by click chemistry-based access to triazole-linked polyUb chains,^174,175^ a bifunctional Ub featuring an internal Boc-protected aminooxy functionality at K6 as well as a C-terminal acetal-caged aldehyde functionality was generated through the combination of amber suppression and intein chemistry (Figure 4d). Simultaneous Boc and acetal deprotection with aqueous trifluoroacetic acid (TFA) followed by oxime ligation afforded K6-linked polyUb chains that were recalcitrant to DUB-catalyzed hydrolysis.^203^

A different strategy for generating Ub-POI conjugates via oxime ligation and GCE was recently developed by the Marx and Scheffner groups. In contrast to earlier approaches, the carbonyl group required for oxime ligation was introduced into the POI in the form of an ncAA, instead of using a C-terminally aldehyde-modified Ub/Ubl variant. This was exemplified by site-specifically incorporating KetoK ((*S*)-2-amino-8-oxononanoic acid, Figure 4e) at position K120 of the tumor suppressor p53. The required aminooxy functionality was accessed by introducing Boc-εOK at position 76 of Ub, enabling the generation of a defined oxime-linked Ub-p53(K120) conjugate upon incubation with p53(K120KetoK) following acidic treatment.^205^ As observed for CuAAC, the addition of SDS in micromolar to millimolar concentrations enhanced oxime ligation and led to increased conjugate formation. Interestingly, it was shown that CuAAC was not suited for the generation of Ub-p53(K120) conjugates, as the zinc binding domain in p53 likely did not tolerate the Cu(I) concentrations in the mM range required for CuAAC.^205^ Importantly, oxime-linked Ub-p53(K120) retained the structural integrity of p53, as shown by binding studies to a cognate DNA sequence and by ubiquitylation assays with specific E3 ligases. As shown for oxime-linked diUbs, oxime-linked Ub-p53(K120) also conferred resistance toward the tested DUBs.^205^

In conclusion, oxime-linked Ub-POI conjugates are excellent structural mimics of natively ubiquitylated proteins. The oxime linkage is a close isostere of the peptide linkage, retaining binding affinities to endogenous binders, such as DUBs and different reader proteins, while at the same time being resistant toward DUB-catalyzed hydrolysis. These combined characteristics make oxime-linked Ub-POI conjugates promising tools for future studies. While neither strategy discussed above explored other donors beside Ub, oxime ligation can in principle be used to generate different Ubl-POI conjugates. Incorporation of Boc-εOK into the acceptor protein limits potential acceptors to simple and refoldable proteins that withstand harsh acidic deprotection. This limitation could be overcome in the future by using photocaged aminooxy functionalities that can be deprotected with light. A corresponding ncAA has already been developed, but the amber suppression yields were significantly lower than those observed for Boc-εOK incorporation.^203^ Directed evolution approaches to yield a more efficient aaRS could solve this problem in the future. Equipping the acceptor protein not with the aminooxy but instead with the carbonyl moiety required for the condensation reaction as shown, *e.g.*, by incorporation of KetoK represents a different strategy to broaden the scope of accessible acceptor POIs.^205^ It should, however, be noted that this approach leads to Ub-POI conjugates displaying linkers that differ markedly in length and composition from the endogenous Ub-POI linkage and have not been analyzed yet in detail from a structural point of view. Compared to the generation of Ub-POI conjugates via CuAAC, oxime ligation strategies tend to be more challenging due to the multistep synthesis of Boc-εOK and obligatory protein refolding. On the other hand, oxime ligation does not depend on Cu(I) and is therefore also applicable for ubiquitylation of proteins that are sensitive toward elevated Cu(I) concentrations.^205^

#### 2.1.3 Thiazolidine Ligation-Based Approaches

**Figure 5:**
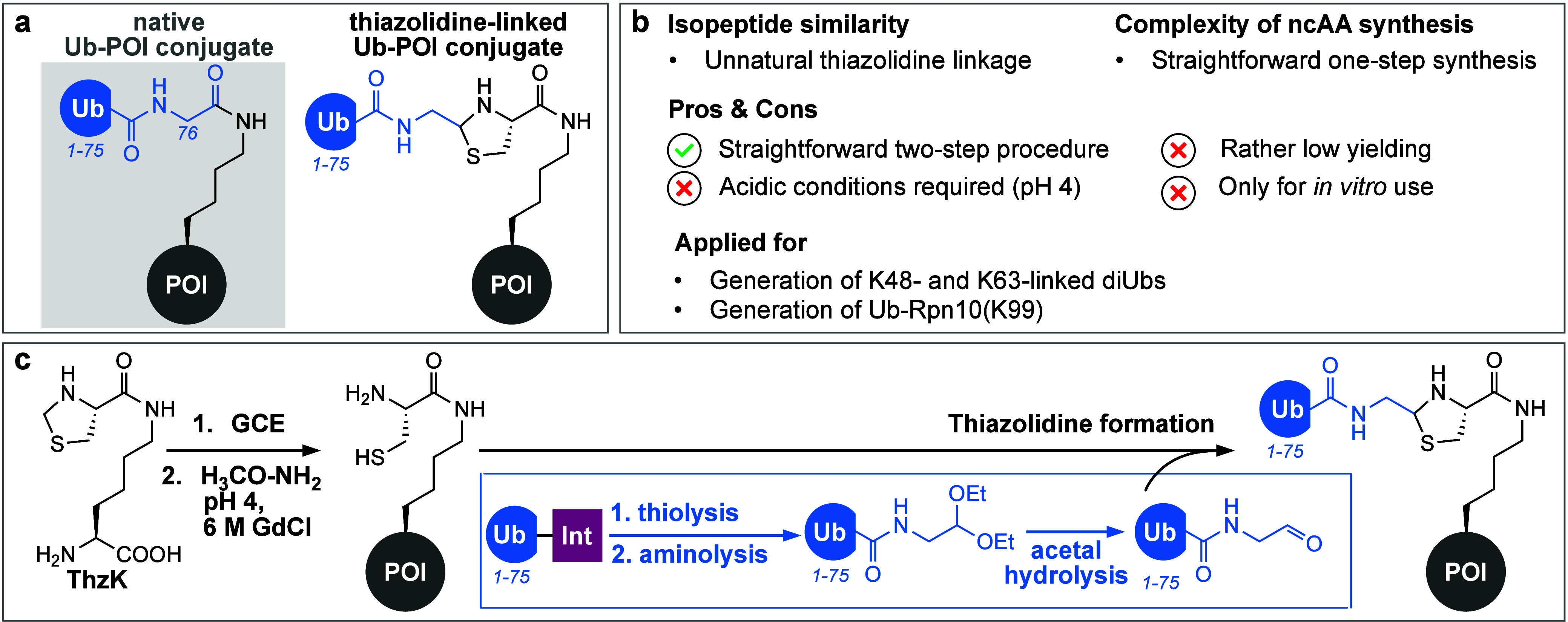
Thiazolidine ligation-mediated ubiquitylation. (a) Schematic representation of a Ub-POI conjugate displaying a thiazolidine linkage. (b) Summary of important characteristics, advantages, disadvantages, and applications of thiazolidine ligation-mediated ubiquitylation. (c) Amber suppression enables site-specific incorporation of ThzK. Subsequent thiazolidine ring cleavage using an acidic cocktail of methoxamine and guanidinium chloride (GdCl) unmasks the reactive 1,2-aminothiol moiety. Ub(1–75) bearing a C-terminal aldehyde moiety is prepared using intein technology. Ligation via thiazolidine ring formation between the 1,2-aminothiol-bearing POI and the Ub-aldehyde results in an artificially linked Ub-POI conjugate.

The bioorthogonal condensation reaction between 1,2-aminothiols and aldehydes to form thiazolidines has been used to generate antibody–drug conjugates and to ligate cyclic peptides by employing N-terminal cysteines as 1,2-aminothiols.^206,207^ GCE has allowed the site-specific incorporation of 1,2-aminothiol moieties, which facilitated bioorthogonal protein labeling using 2-cyanobenzothiazole-modified fluorophores^208^ as well as protein ubiquitylation via NCL (native chemical ligation, see section 2.2.2).^209^ Liu and co-workers exploited this reaction for the generation of thiazolidine-linked Ub-POI conjugates (Figure 5a and b) through the site-specific incorporation of ThzK (*N*ε-thiaprolyl-l-lysine, Figure 5c), a lysine derivative bearing a thiaprolyl moiety, via GCE.^210^ Acidic methoxyamine treatment^211^ of ThzK-containing proteins under denaturing conditions installs a 1,2-aminothiol moiety on the POI. A thiazolidine-bridged Ub-POI conjugate is afforded upon reaction with an intein-derived Ub that is modified with a C-terminal aldehyde^191^ (Figure 5c).^210^

The method was applied for the generation of thiazolidine-linked K48- and K63-diUbs, as well as for the monoubiquitylation of the proteasomal subunit Rpn10 at position K99. So far, however, the structural integrity and binding capabilities of these conjugates toward DUBs have not been investigated. In its current implementation, the method suffers from rather limited yields (10–30%) and the requirement of acidic and denaturing conditions needed for generating the reactive 1,2-aminothiol moiety, restricting this technology to very stable and easily refoldable proteins.

### 2.2 Strategies Resulting in Ub-POI Conjugates with Native Linkages

While the previously discussed approaches result in artificially linked Ub-POI conjugates, considerable effort has been undertaken in the past decade in the development of methodologies to access isopeptide-linked conjugates to better mimic natively ubiquitylated proteins. Amide-bond-yielding bioorthogonal reactions^60^ include the traceless Staudinger ligation between azides and phosphines^212−214^ and ligation reactions between hydroxyl amines and α-keto acids, acylboronates, or acylsilanes (KAHA, KAT, MIDA, or ASHA ligations, respectively).^215−217^ Staudinger ligation between AznL (azidonorleucine), a lysine derivative bearing an ε-azide group that can be incorporated in response to an amber codon using a PylRS variant^218^ or a methionine codon employing a modified MetRS,^66^ and a Ub C-terminal phosphino-thioester has conceptually been contemplated but so far has not been used to access Ub-POI conjugates.^48,219,220^ Possible challenges include the slow kinetics of the Staudinger ligation as well as the susceptibility of phosphino-thioesters to oxidation. Hydroxylamine ligations with α-keto acids or acylboronates might present further attractive possibilities to access defined Ub-POI conjugates.^221^ The corresponding functionalities have, however, not yet been incorporated into proteins via GCE.

As an alternative, complex protection/deprotection protocols to cage all but one lysine residue within both the target protein and Ub/Ubl have been developed and used for controlled chemical formation of an isopeptide bond between POI and Ub/Ubl, followed by deprotection of all lysine-caging groups (section 2.2.1). Furthermore, researchers have utilized nature’s functional repertoire and have adapted both inteins (section 2.2.2) and certain classes of transpeptidases (section 2.2.3) to react selectively with site-specifically installed noncanonical moieties to form isopeptidically linked Ub-POI conjugates. These approaches are described in detail below.

#### 2.2.1 Genetically Encoded Orthogonal Protection and Activated Ligation (GOPAL)

**Figure 6:**
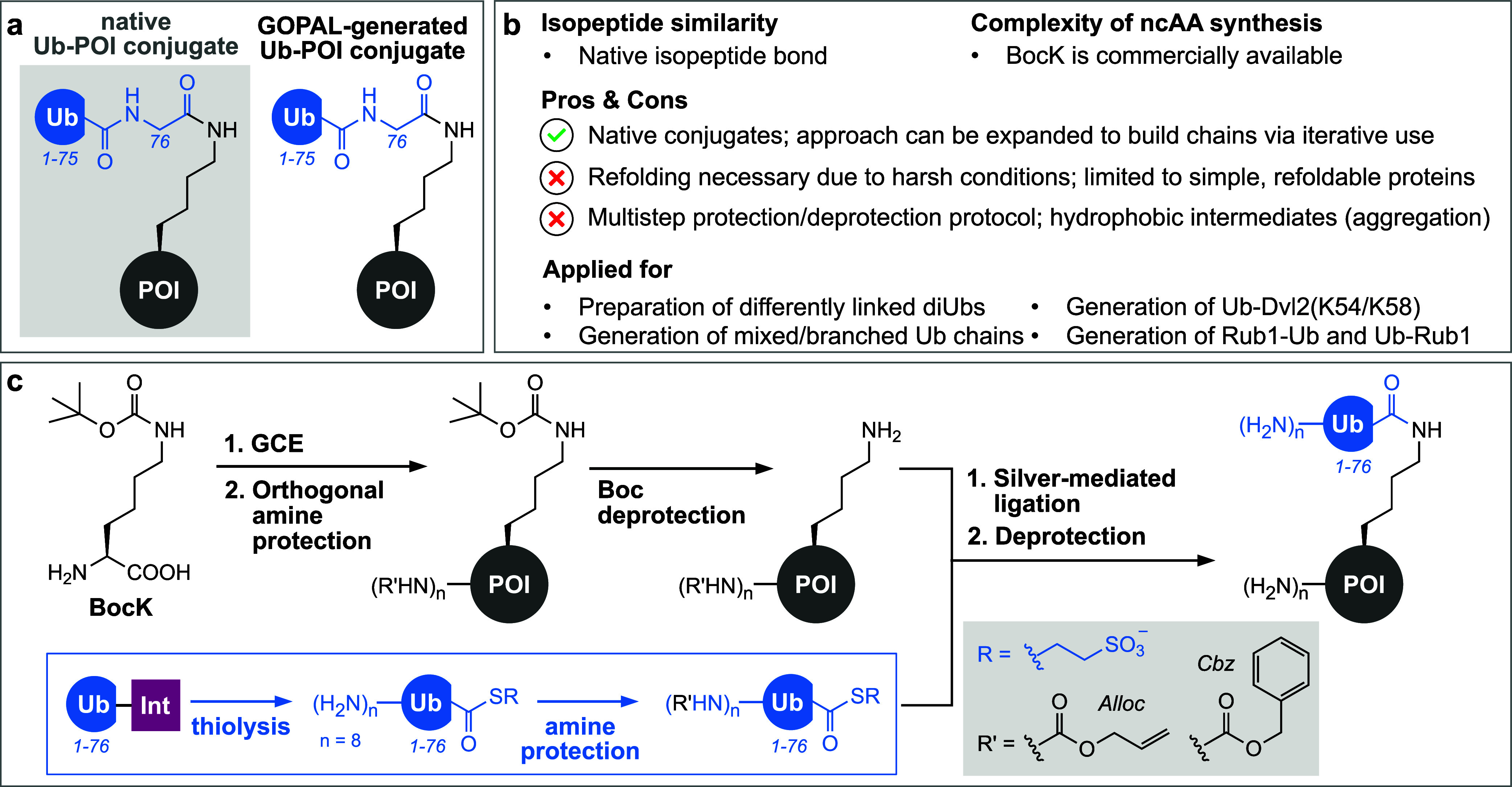
Ubiquitylation using GOPAL. (a) Schematic representation of a natively linked Ub-POI conjugate. (b) Summary of important characteristics, advantages, disadvantages, and applications of the GOPAL strategy. (c) BocK is site-specifically incorporated into the POI using amber suppression, which is followed by global protection of amine moieties with the Cbz or the Alloc protecting group. Acidic treatment using TFA facilitates Boc deprotection, resulting in a POI with a single reactive amine moiety. In parallel, global amine protection with the Cbz or the Alloc protective group of an intein-derived Ub(1–76)-thioester is performed. Silver-mediated ligation between the POI, bearing a single reactive lysine residue, and the globally protected Ub-thioester results in the formation of a native isopeptide bond between the Ub and the POI. Global deprotection to remove all Cbz or Alloc groups yields native Ub-POI conjugates.

To build Ub-POI conjugates with native isopeptide linkages, Chin and co-workers developed a protection/deprotection strategy dubbed GOPAL (genetically encoded orthogonal protection and activated ligation, Figure 6a and b).^222^ The striking difference between GOPAL and the approaches described above is that GOPAL does not require any bioorthogonal reaction handle (*e.g.*, azide, alkyne, aminooxy, or 1,2-aminothiol) on the acceptor protein but instead works by protecting all but one lysine residue in the donor and acceptor proteins to enable specific Ub conjugation at this one lysine residue. For this, ncAA BocK (*N*ε-(*tert*-butoxycarbonyl)-l-lysine, Figure 6c) is incorporated site-specifically at the target lysine position within the acceptor POI using the wt *Mb*PylRS/tRNA pair, followed by subsequent installation of an orthogonal protecting group on all remaining free primary amines of the POI (all lysine residues and the N-terminus). Subjecting the BocK-bearing POI to Cbz-OSu (*N*-(benzyloxycarbonyloxy)succinimide) treatment leads to modification of the protein N-terminus as well as the free lysine side chains with Cbz (benzyloxycarbonyl) groups. Subsequent acidic on-protein Boc deprotection (typically with 60% TFA) affords an acceptor POI that presents a single reactive primary amine at the chosen conjugation site. To access a donor Ub suitable for GOPAL, an intein-derived C-terminal Ub-thioester is globally protected with Cbz-OSu and converted *in situ* to an activated *N*-hydroxysuccinimidyl ester in the presence of Ag(I).^223,224^ Combination of the activated donor and the acceptor POI bearing a single unprotected lysine residue affords selective isopeptide bond formation. After successful ligation, global Cbz deprotection with an acidic cleavage cocktail^225^ followed by protein refolding yields a natively linked Ub-POI conjugate (Figure 6c).^222^ GOPAL enabled for the first time the generation of atypically linked diUbs such as K6- and K29-linked diUbs. These diUbs were used for the structural elucidation of K6-diUb as well as for the characterization of both linkages in quantitative DUB assays that revealed the high activity of the OTU-type DUB TRABID toward the K29 linkage.^222^ Further in-depth profiling of TRABID in a follow-up study that investigated all eight possible diUb linkages confirmed the K29-specificity of TRABID and discovered additional activity of TRABID toward K33-linked diUbs.^226^ GOPAL-generated diUbs were furthermore applied to develop a MALDI-TOF-based DUB screening assay.^227^ The initial concept of GOPAL was further expanded by the use of an Alloc (allyloxycarbonyl) protection group instead of the Cbz protection group for global amine protection (Figure 6c).^228^ Choosing the Alloc protection group over the Cbz group proved advantageous as its deprotection conditions, involving catalytic amounts of Ru(II) complexes in the presence of an excess of thiophenol, are milder than the acidic cocktail used for Cbz deprotection and are completely orthogonal to the employed Boc deprotection conditions. This enabled the sequential application of GOPAL for accessing, apart from all differently linked diUbs,^229^ more complex Ub chain topologies, such as homotypic,^228,230,231^ and branched Ub chains.^232^ Ub chains generated via GOPAL were used in studies employing NMR and top-down mass spectrometry that contributed to a better structural understanding of various Ub topologies.^229−234^

As GOPAL requires multiple steps of chemical protection and deprotection as well as subsequent protein renaturation, it is generally restricted to proteins that can be easily refolded. Nevertheless, GOPAL was also used to generate Ub-POI conjugates beyond Ub chains. The yeast homologue of NEDD8, Rub1,^235^ was used both as donor as well as acceptor in GOPAL-derived Rub1-Ub and Ub-Rub1 conjugates in a study by Fushman and co-workers.^236^ Three hybrid chains that were previously observed under cellular stress conditions,^237,238^ Rub1-Ub(K29), Rub1-Ub(K48), and Ub-Rub1(K48), were generated via GOPAL and subjected to biochemical analysis, which revealed the derubylase activity of USP5 and OTUB1.^236^ In a different study, GOPAL was used for the ubiquitylation of the DIX domain of Dvl2 (dishevelled 2), a eukaryotic protein that plays an important role in Wnt signaling.^239^ The dynamic head-to-tail polymerization of the DIX domain of Dvl2 is essential for its interaction with Axin and therefore for the Wnt signaling activity of Dvl2.^240^ Biochemical studies uncovered that ubiquitylation of the DIX domain at K54 blocks head-to-tail polymerization, while ubiquitylation at K58 does not influence polymerization activity, rendering polymerization of the DIX domain dependent on the ubiquitylation status of K54. DUB profiling using GOPAL-derived Ub-DIX conjugates identified 28 active DUBs, including cezanne and CYLD, that specifically hydrolyze Ub-DIX(K54) while not acting on Ub-DIX(K58).^239^

In conclusion, GOPAL provided for the first time access to atypical Ub chains linked via native isopeptide bonds. This paved the way for diverse structural studies and DUB profiling of different Ub topologies. The scope of POIs suitable for ubiquitylation via GOPAL is, however, limited to stable and easily refoldable proteins, as the multistep approach requires quite harsh protection/deprotection procedures and protein renaturation. Furthermore, the fully Cbz- or Alloc-protected acceptor proteins present highly hydrophobic intermediates that are incompatible with protein solubility, tend to aggregate, and can be very difficult to handle and purify.^222^ Since its first description, further strategies based on the GOPAL concept to globally and iteratively protect and deprotect natural amino acid residues and thereby single out one individual and reactive natural amino acid within a target protein have been further adapted to allow the generation of Ub-POI conjugates linked via thioether bonds, as described in more detail below,^241^ or native amide bonds.^242^

**Figure 7:**
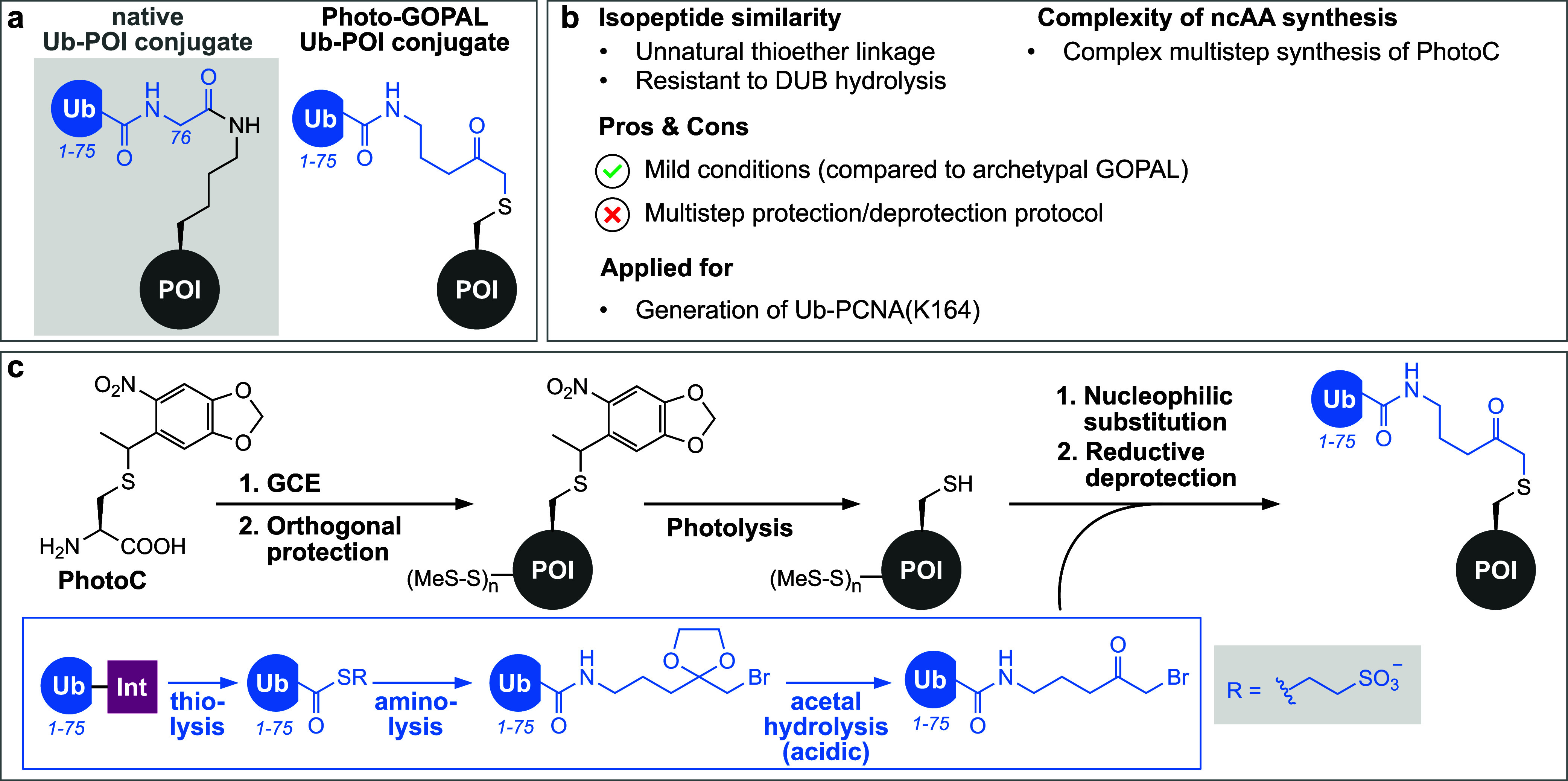
Ubiquitylation using Photo-GOPAL. (a) Schematic representation of a thioether-linked Ub-POI conjugate. (b) Summary of important characteristics, advantages, disadvantages, and applications of the Photo-GOPAL strategy. (c) PhotoC is site-specifically incorporated into the POI using amber suppression, which is followed by global protection of thiol moieties with MMTS (*S*-methylmethanethiosulfonate). Photolysis of PhotoC affords a POI with a single reactive thiol moiety. Intein technology provides access to a donor-Ub equipped with a C-terminal α-bromoketone moiety that undergoes a nucleophilic substitution reaction with the sole unprotected thiol in the acceptor POI, yielding a thioether linked Ub-POI conjugate. Subsequent global MMTS deprotection is performed under reducing conditions.

A GOPAL-inspired approach that results—in contrast to archetypal GOPAL—in DUB-resistant non-natively linked Ub-POI conjugates is based on the site-specific incorporation of a photocaged cysteine (PhotoC) at acceptor lysine sites within the target protein via amber suppression^241^ using a previously reported specific PylRS/tRNA variant^243^ (Figure 7a and b). Conversion of the remaining native cysteines within the POI into disulfides using MMTS (*S*-methylmethanethiosulfonate) followed by orthogonal photodeprotection of the genetically encoded PhotoC via irradiation at 365 nm results in a target protein with only one reactive thiol moiety (Figure 7c). The complementary donor Ub, bearing a cysteine-reactive moiety at its C-terminus, is prepared via thiolysis of a Ub-intein fusion with an α-bromoketone-containing linker that can undergo a nucleophilic substitution reaction with the deprotected cysteine within the POI. Subsequent reductive cleavage of MMTS-derived disulfides generated Ub-POI conjugates that are linked via a noncleavable thioether bond (Figure 7a). This methodology enabled the ubiquitylation of PCNA at K164. Furthermore, studies on Polη, including translesion synthesis assays as wells as ATPase assays with RFC (replication factor C), showed that the photo-GOPAL-derived Ub-PCNA(K164) behaves similarly to its native counterpart. Since the advantages of photo-GOPAL over archetypal GOPAL involve the rather mild conditions used for orthogonal protection and deprotection (light and disulfide formation/cleavage), photo-GOPAL does not require refolding of the Ub-POI conjugate. The disadvantages, on the other hand, include the creation of an artificial linkage between Ub and POI, the requirement of oxidative conditions for disulfide-mediated protection, and the challenge of achieving complete disulfide protection while preventing potential disulfide exchange after light-mediated deprotection (Figure 7b). Additionally, *ortho*-nitrobenzylic photocleavable protection groups were shown to be metabolically labile within the bacterial cytoplasm, which may lead to diminished yields.^244^

#### 2.2.2 Native Chemical Ligation-Based Approaches

A versatile and robust method for the ligation of unprotected peptides and proteins resulting in an amide bond is represented by NCL (native chemical ligation).^245,246^ NCL requires a C-terminal thioester on the N-terminal peptidic fragment and a 1,2-aminothiol moiety presented by an N-terminal cysteine on the C-terminal peptidic fragment. Mechanistically, NCL involves first a nucleophilic attack of the thiol moiety of the N-terminal cysteine onto the C-terminal thioester. The product of this *trans*-thioesterification reaction spontaneously rearranges via an S–N-acyl-shift that results in an amide bond. Accordingly, products of NCL inherently feature a cysteine at the ligation site, which limits the possibilities for scarless ligations. To address this constraint, desulfurization procedures that convert cysteine to the much more abundant alanine after NCL were developed,^247,248^ although it should be noted that such desulfurization procedures obviously target all cysteines within a protein sequence. Nevertheless, accompanied by advances in SPPS and peptide or protein thioester synthesis, NCL and EPL have facilitated the semisynthesis of many proteins over the last 30 years, including proteins bearing site-specific modifications, such as PTMs, d-amino acids, and biophysical probes.^249^

This development did not go unnoticed by chemical biologists working in the Ub field and inspired several approaches based on site-specific installation of a 1,2-aminothiol moiety within the POI. In combination with intein chemistry-derived C-terminal Ub-thioesters, NCL can be employed to generate Ub-POI conjugates. Muir and co-workers pioneered the approach with the development of a strategy for the site-specific ubiquitylation of histone H2B. The rather sophisticated procedure employed two subsequent NCL reactions. First, an intein-derived C-terminal Ub-thioester was reacted with a peptide corresponding to the C-terminus of H2B(117–125) that carried both a photocaged N-terminal cysteine and, at the acceptor lysine position (K120), a lysine modified with a photoprotected thiol-based ligation auxiliary mimicking the 1,2-aminothiol moiety.^250^ The resulting natively ubiquitylated H2B peptide was, after photodeprotection, susceptible to a second NCL with the thioester-modified N-terminal part of H2B(1–116). Desulfurization to the native A117 and *in vitro* reconstitution of nucleosomes bearing Ub-H2B(K120) allowed biochemical analyses of site-specifically ubiquitylated nucleosomes.^251^ Subsequent improvements of this approach introduced either a 1,3-aminothiol or a 1,2-aminothiol group within the acceptor lysine side chain by adding a sulfhydryl group to the γ- or δ-carbon (resulting in γThioK and δThioK), respectively. This advancement allowed access to various ubiquitylated peptides and chemically synthesized proteins,^46^ including monoubiquitylated^252,253^ and polyubiquitylated^254^ peptides, diUbs,^255,256^ K48-linked tetraUb,^257^ tetraubiquitylated α-synuclein^258^ and ubiquitylated α-globulin.^259^

**Figure 8:**
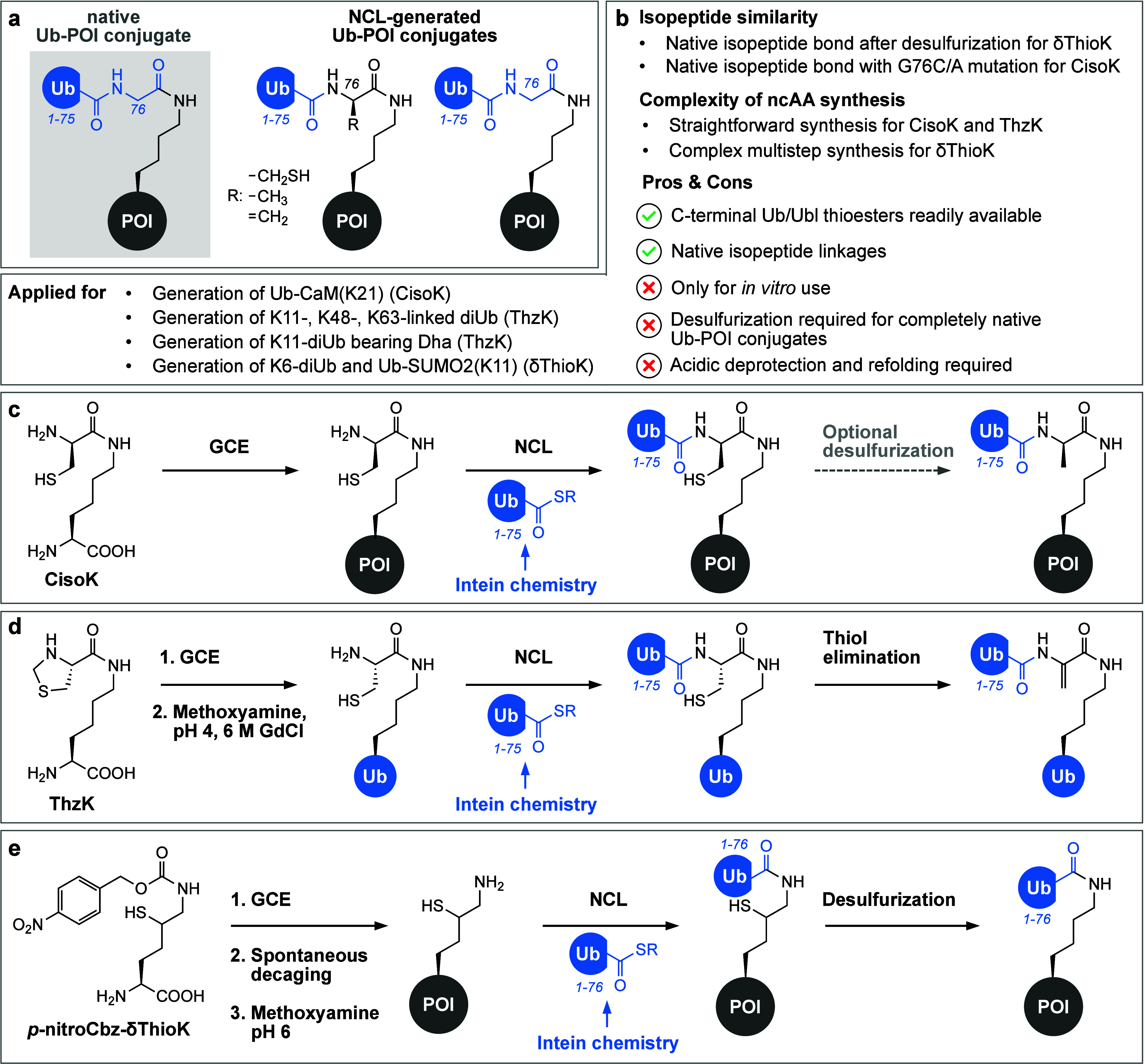
NCL-mediated ubiquitylation. (a) Schematic representation of NCL-derived Ub-POI conjugates as described in subpanels c and d (left) or e (right). (b) Summary of important characteristics, advantages, disadvantages, and applications of NCL-based strategies. (c) CisoK is site-specifically incorporated into the POI using amber suppression. Subsequent NCL with a intein-derived Ub(1–75)-thioester yields a Ub-POI conjugate with a native isopeptide bond and a G76C mutation. Optional desulfurization would result in a conjugate with a G76A mutation instead of a G76C mutation. (d) Site-specific incorporation of ThzK into Ub followed by treatment with methoxyamine at acidic pH unmasks a 1,2-aminothiol moiety that can undergo NCL with Ub(1–75)-thioester. Thiol elimination converts C76 to Dha, yielding an activity-based probe. (e) Site-specific incorporation of *p*-nitroCbz-δThioK using amber suppression followed by NCL with an Ub(1–76)-thioester and subsequent desulfurization yields a completely native Ub-POI conjugate.

Strategies based on chemical peptide and protein synthesis are, however, limited to the ubiquitylation of peptides and/or to ubiquitylation sites close to protein termini and furthermore require refolding of the Ub-POI conjugates. Although it is possible to modify intermediate sites, this necessitates arduous multistep ligations. Accordingly, endeavors to genetically encode 1,2-aminothiol moieties have been undertaken in order to expand the scope of potential target proteins (Figure 8a and b). The first efforts by Chan and co-workers to incorporate the 1,2-aminothiol moiety in the context of an ncAA succeeded by simply coupling cysteine to the ε-amino group of lysine via an isopeptide bond (CisoK, Figure 8c).^209^ Both diastereomers of CisoK, with d-cysteine or l-cysteine attached to the Nε of l-lysine, were successfully incorporated into proteins using the wt *Mb*PylRS/tRNA pair. Nevertheless, only the d-cysteine coupled diastereomer was used for further experiments due to better incorporation efficiencies. Incubation of CaM (calmodulin) bearing CisoK at position K21 with intein-derived Ub(ΔG)-thioester yielded the corresponding Ub-CaM(K21) conjugate, displaying a native isopeptide bond and a d-cysteine residue instead of G76 at the Ub C-terminus in approximately 30% yield. In agreement with previous studies based on enzymatically derived Ub-CaM conjugates,^260^ the NCL-generated Ub-CaM(K21) conjugate was shown to decrease the activity of phosphorylase kinase compared to unmodified CaM.^260^ Unfortunately, NCL-derived Ub-POI conjugates bearing the G76d-C mutations were not probed in DUB assays and therefore no information is available regarding their stability toward proteolysis by DUBs.

An alternative approach presented by Yin and co-workers consists of incorporating ThzK (Figure 5c), followed by treatment with methoxyamine at acidic pH to open the thiazolidine ring and access CisoK (Figure 8d). Combined with a donor Ub(ΔG)-thioester, this method was used to generate K11-, K48-, and K63-linked diUbs via NCL.^261^ The G76C-bearing linked diUbs were shown to form mixed disulfides with the active site cysteine of the E2 enzyme UBE2S (K11-linked diUb) or with single-site cysteine E3 mutants of the HECT domains of HUWE1 (K48-diUb) and NEDD4 (K63-diUb) in the presence of 5,5′-dithiobis(2-nitrobenzoic acid). The specificity of this disulfide trapping was, however, not investigated in more detail. The K11-diUb was further subjected to the conversion of C76 in the linker region to dehydroalanine (G76Dha) by treatment with α,α′-dibromoadipyl(bis)amide. This thiol elimination afforded a DUB probe that covalently reacted with OTUB1 as well as OTUB2 and to a lesser extent with USP2.^261^

Another approach combining genetic code expansion and NCL to generate Ub-POI conjugates entails site-specific installation of the above-described δThioK (δ-thiolysine), which was first developed by Brik and colleagues for making Ub conjugates of SPPS-derived peptides.^252^ For selective recognition of δThioK by a PylRS mutant and to avoid mischarging by the lysyl-tRNA synthetase, Chin and co-workers designed and synthesized an ncAA that bears—apart from the δ-thiol moiety—a *p*-nitro-Cbz protection group at the *N*ε-amino group of *p*-nitroCbz-δThioK (δ-thiol-*N*ε-(*p*-nitrocarbobenzyloxy)-l-lysine, Figure 8e).^204^ This caging group is removed post-translationally, either *in cellulo* or during cell lysis, by the reduction of the nitro group and concomitant elimination of the corresponding imine, which is triggered by the expulsion of CO_2_, making additional deprotection steps obsolete. Interestingly, MS analysis indicated that site-specific incorporation of *p*-nitroCbz-δThioK into a POI did not lead to δThioK-modified proteins but instead to a thiazolidine adduct resulting from a condensation reaction of the 1,2-aminothiol moiety with cellular pyruvate. In a further deprotection step with methoxyamine under slightly acidic conditions (pH 6), the thiazolidine adduct could be cleaved to reinstate the NCL-reactive 1,2-aminothiol functionality. Using this procedure, Ub(K6δThioK) was generated and incubated with intein-derived Ub-thioester to access the corresponding diUb via NCL (Figure 8e). K6-diUb, bearing δThioK at the ligation site, was then desulfurized using free radical-based reduction^248^ followed by refolding to access completely native diUb. Circular dichroism spectroscopy and DUB screening assays with USP2 and USP5 confirmed the biological integrity of NCL-derived K6-diUb and the expansion of the approach to the ubiquitylation of SUMO2 at position K11, which resides within the disordered N-terminal domain, demonstrating the applicability toward a non-Ub target protein.^204^ Access to this Ub-SUMO conjugate revealed that the DUB UCHL3 readily cleaves Ub from the prepared conjugate, revealing that UCHL3 might be tasked with removing Ub from intrinsically disordered protein regions in the cell.^262^

In conclusion, ubiquitylation using CisoK and NCL is straightforward as the ncAA is easily accessible. Nevertheless, the approach does not result in a completely native conjugate but rather in an isopeptide-linked Ub-POI conjugate bearing a d-cysteine at position 76 in the donor Ub. Desulfurization of d-cysteine to d-alanine may, however, be attempted using different available protocols.^263,264^ Furthermore, information concerning the structural and functional properties of these Ub-POI conjugates as DUB substrates is lacking. Conversion of d-cysteine at this position to Dha can, however, be used to generate Ub-POI conjugates that bear an electrophilic warhead and can covalently react with DUBs. Obviously, this is only possible for Ub-POI conjugates that do not contain any other cysteine residues. NCL between δThioK-bearing proteins and an Ub-thioester allows the production of completely native conjugates after a free radical-based desulfurization step. Synthesis of the employed *p*-nitroCbz-δThioK, on the other hand is far more complex than the synthesis of CisoK or ThzK. The 1,2-aminothiol moieties (both in CisoK and δThioK) are not inert under physiological conditions inside cells, as they react with electrophilic metabolites such as pyruvate and likely also α-ketoglutarate to the corresponding thiazolidine adducts that have to be cleaved under slightly acidic conditions with methoxyamine, restricting the approach to stable and refoldable proteins. Similar limitations are posed by the desulfurization step that requires denaturing conditions. Accordingly, the described approaches based on a combination of GCE and NCL have only been used for Ub and SUMO as target proteins.

#### 2.2.3 Ubiquitylation and SUMOylation through Chemoenzymatic Methods

##### 2.2.3.1 Sortase-Mediated Ubiquitylation and SUMOylation

**Figure 9:**
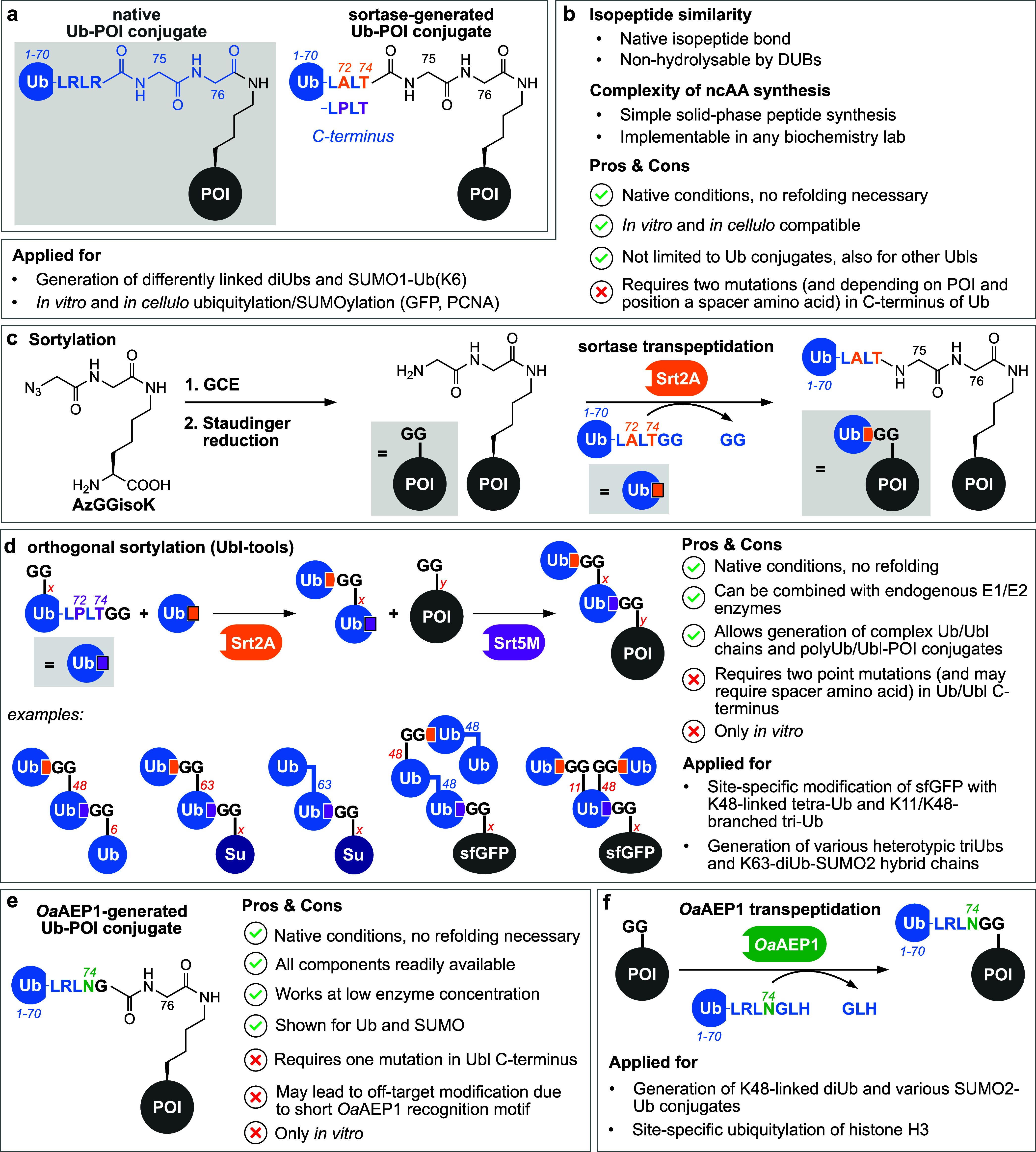
Chemoenzymatic ubiquitylation strategies. (a) Schematic representation of a sortase-generated Ub-POI conjugate displaying a native isopeptide bond and two point mutations in the flexible Ub C-terminus. (b) Summary of important characteristics, advantages, disadvantages, and applications of sortylation. (c) Amber suppression allows the site-specific incorporation of AzGGisoK into proteins. AzGGisoK-bearing POIs can be converted to GGisoK-bearing POIs via biorthogonal Staudinger reduction *in vitro* and *in cellulo*. By introducing two point mutations into the flexible Ub C-terminus (Ub-AT), a sortase recognition motif is installed within the donor Ub C-terminus. Sortase-mediated transpeptidation between Ub-AT and the GGisoK-bearing POI yields a Ub-POI conjugate displaying a native isopeptide bond and two point mutations in the Ub C-terminus. (d) Schematic overview of various Ubl topologies afforded by iterative use of orthogonal sortases and important characteristics and applications thereof. (e) Schematic representation of an *Oa*AEP1-generated Ub-POI conjugate displaying a native isopeptide bond and one point mutation in the flexible Ub C-terminus. (f) Site-specific incorporation of AzGGisoK followed by Staudinger reduction yields GGisoK-bearing POIs that can be employed in a *Oa*AEP1-mediated transpeptidation reaction with Ub that features one point mutation in the C-terminus.

To expand the methods for generating Ub/Ubl-POI conjugates linked by native isopeptide bonds to various target proteins, including complex and nonrefoldable proteins, and to ideally also trigger specific ubiquitylation and SUMOylation events in living cells, our group recently developed a chemoenzymatic approach, dubbed sortylation (Figure 9a–c).^265^ As the name might indicate, sortylation involves the use of sortase A, a transpeptidase from *Staphylococcus aureus* that is responsible for modifying the bacterial cell wall by anchoring proteins to the peptidoglycan layer on Gram-positive bacteria. Since their first characterization in 1999,^266^ sortases have been widely used in protein semisynthesis as well as in protein engineering approaches to decorate protein termini with a variety of different functionalities such as fluorophores, oligonucleotides, metal chelators, and click handles.^267−269^ Sortase enzymes catalyze the transpeptidation reaction between a five amino acid recognition motif within their target protein, *e.g.*, LPXTG (where X represents any amino acid), and a polyglycine substrate in a two-step process. In a first step the active site cysteine of sortase attacks the peptide bond between T and G in the recognition motif, thereby cleaving the bond and forming a thioester intermediate. Subsequently, this thioester is nucleophilically attacked by the N-terminal amine of a polyglycine substrate, leading to the formation of a native peptide bond. By means of directed evolution, sortase A enzymes with specificities for recognition sequences other than the LPXTG motif can be obtained. Researchers identified sortase variants that specifically recognize and cleave LAXTG (Srt2A),^270^ LPXSG (Srt4S),^270^ APXTG (SrtF40),^271^ FPXTG (SrtF21),^272^ HPXTG (SrtW54),^273^ and LMVGG (SrtAβ)^274^ motifs. In order to make use of sortase for the generation of Ub-POI conjugates, a closer look at the Ub C-terminus is necessary. Ub is highly conserved throughout eukaryotic evolution, and the unstructured C-terminus displays the sequence LRLRGG.^275^ It is therefore possible to place a sortase recognition motif, *e.g.*, the recognition motif for Srt2A, within the Ub C-terminus by introducing two point mutations into the LRLRGG motif (*i.e.*, R72A and R74T), thereby creating Ub-AT bearing the C-terminus LALTGG (Figure 9a and c). To attach this Ub-AT variant to a target protein, our group designed and synthesized the ncAA GGisoK, in which a diglycine motif is attached via an isopeptide bond to the *N*ε of lysine, and showed that sortase catalyzes transpeptidation between Ub-AT and GGisoK.^265^ Furthermore, by masking the terminal amino moiety of the diglycine motif in GGisoK as an azide, generating the ncAA AzGGisoK (Figure 9c), sortylation can be rendered inducible by the addition of a small molecule that reduces the azide in AzGGisoK to an amine (GGisoK). Directed evolution approaches using custom-designed PylRS libraries resulted in a novel PylRS variant with selective recognition of AzGGisoK, enabling site-specific incorporation of AzGGisoK into target proteins. AzGGisoK-modified proteins were readily reduced to the corresponding GGisoK-bearing proteins via *in cellulo*-compatible Staudinger reduction using triphenylphosphine derivates such as 2DBPA (2-(diphenylphosphino)benzoic acid), which has been shown to be applicable in living prokaryotic and eukaryotic cells.^276^ Upon phosphine-triggered reduction, the resulting GGisoK-modified proteins can participate in Srt2A-mediated transpeptidation with Ub-AT to yield site-specifically ubiquitylated target proteins connected via a native isopeptide linkage and displaying the two point mutations R72A and R74T in the linker region between Ub and POI (Figure 9c). The efficiency and kinetic performance of sortylation are dependent on the steric accessibility of the recognition motif within Ub-AT, and both reaction rate and yield can be increased by introducing leucine as spacer amino acid between the C-terminal ß-sheet of Ub and the first amino acid of the sortase recognition motif, generating Ub-LAT with a C-terminal LLALTGG sequence. This shifts the sortagging motif one amino acid away from the compact Ub ß-grasp fold, which improves the access of sortase to the Ub C-terminus. *In vitro* sortylation allowed the generation of differently linked diUbs as well as site-specific ubiquitylation of PCNA at K164. DUB assays with Srt2A-generated diUbs and Ub-PCNA(K164) revealed that the two point mutations within the Ub C-terminus confer resistance toward DUB hydrolysis. To validate the structural and physiological properties of sortase-generated diUbs, binding assays with various UBDs were conducted. Pull-down assays using the NZF (Npl4 zinc finger) domain of kinase TAK1 confirmed that Srt2A-generated K63-diUbs (both with and without spacer leucine in the linker region) retained the ability to bind the TAK1-NZF domain.^277^ A similar behavior was observed for the recognition of sortylation-derived K48-linked diUbs by the proteasomal shuttling factor hHR23A(human homologue of yeast Rad23a)-UBA2.^278^ Furthermore, fluorescence anisotropy experiments uncovered similar binding constants of natively linked K63-diUb and Srt2A-generated K63-diUbs toward Rap80 (receptor associated protein 80), which targets BRCA1 (breast cancer type 1 susceptibility protein), to DNA damage loci via its tandem UIM by recognizing K63-diUb.^279^ Additionally, MD simulations with K48- and K63-diUbs bearing *in silico*-modeled AT or LAT linkers confirmed that the mutations did not lead to structural rearrangements and did not show altered diUb dynamics. These experiments indicate that sortase-generated diUbs, despite being resistant toward DUB cleavage, retain their binding affinity to linkage-specific UBDs, a requirement for triggering various signaling pathways, including protein degradation, DNA-damage repair, and protein kinase activation.^265^

Since all Ubls share the common ß-grasp fold and importantly display a flexible unstructured C-terminus, sortylation can be expanded toward Ubls by simply introducing the sortagging motif via one or two point mutations in the Ubl C-terminus. This was demonstrated for SUMOylation, which was accessible through mutation of the native SUMO1 C-terminus QEQTGG to LAQTGG, enabling the site-specific SUMOylation of target proteins in *in vitro* experiments.^265^

Sortylation is a chemoenzymatic reaction that works under native conditions, allowing the attachment of Ubls to complex, multimeric, nonrefoldable proteins, as exemplified by the site-specific ubiquitylation and SUMOylation of the trimeric protein PCNA. The chemoenzymatic nature of the approach also enabled for the first time artificial and site-specific ubiquitylation and SUMOylation of proteins within living cells. Sortase, generally a Ca^2+^-dependent enzyme, can be engineered by introducing two point mutations to work in surroundings without or with very low Ca^2+^ concentrations, such as the cytosol of living cells.^280^ By overexpressing the Ca^2+^-independent sortase variant, Ub-AT, as well as a POI bearing AzGGisoK, site-specific ubiquitylation and SUMOylation of target proteins can be chemically triggered,^265^ providing a useful tool for producing ubiquitylated/SUMOylated eukaryotic proteins in the established workhorse *E. coli*.^281^

For the expansion of sortylation toward eukaryotes, first, site-specific incorporation of AzGGisoK into mammalian proteins was successfully established in mammalian cell culture using the orthogonal AzGGisoKRS/tRNA pair. To establish sortylation as a ubiquitylation approach that is orthogonal to and independent of the endogenous E1/E2/E3 machinery, Ub variants bearing the LLALTG Srt2A recognition motif followed by a rigid six amino acid sequence were designed.^265^ These Ub variants lack the highly conserved C-terminal GG motif of Ub, and their overexpression in mammalian cells confirmed that they are not recognized and charged by E1, E2, and E3 enzymes, allowing sortylation to happen in parallel and independently of endogenous Ubl modification. Cotransfection of mammalian cells with plasmids coding for these Ub-variants together with Srt2A confirmed lack of misubiquitylation of endogenous proteins by sortase and the successful site-specific ubiquitylation of AzGGisoK-bearing proteins after *in vivo* Staudinger reduction via the addition of 2DPBA to the growth media. Using this approach, site-specific ubiquitylation and SUMOylation of sfGFP at position N150 and PCNA at K164 in living cells were achieved.^265^

Taken together, sortylation is an unprecedented chemoenzymatic approach to ubiquitylate and SUMOylate proteins *in vitro* and in living cells and may represent a promising tool to further dissect the complex Ub/Ubl system. Fine-tuning of all needed components renders *in cellulo* sortylation independent of and orthogonal to the endogenous machinery, yielding Ub-POI conjugates that are resistant toward hydrolysis by DUBs and allows the study of the effects of stable Ub-POI conjugates in physiological settings. Importantly, sortase-generated Ub-POI conjugates retain the important surface areas and hydrophobic patches within Ub, and interactions with UBDs remain intact, establishing valuable tools for interrogating cell signaling pathways and assigning Ub-specific proteins.

The ease of use of this three-component sortylation system allows facile access to defined Ub/Ubl-POI conjugates within any biology lab, as the amino acid AzGGisoK can be readily synthesized on multigram scale via SPPS and the other components (Ubl, sortase) do not require any chemical manipulation. Additionally, sortylation may be expanded into a tool for identifying Ub/Ubl-binding proteins and for profiling substrate-specific DUBs and E3 enzymes by introducing photocrosslinking and proximity-triggered crosslinking functionalities into Ub/Ubl.^282,138,283^ Furthermore, sortylation may allow the site-specific direction of otherwise nonviable Ub mutants to POIs, enabling structure–activity studies that employ Ub mutants or analogues.^284^ Recent findings have shown that Ub itself can be modified by other PTMs, including through acetylation and phosphorylation (see section 3).^44^ Sortylation, combined with GCE tools to site-specifically incorporate phosphorylated or acetylated amino acids, will allow the generation of defined Ub-POI conjugates in which Ub bears further PTMs, thereby providing a tool to study mechanistic effects of Ub PTMs and find specific reader proteins for these complex modifications. A potential shortcoming of sortylation consists of the need to introduce two point mutations into the Ub/Ubl-C-terminus. Even though several assays have shown that sortase-generated Ub/Ubl-POI conjugates retain their structural dynamics and biological functions, it is not possible to completely generalize this outcome. Interestingly, sortase enzymes can be engineered to recognize different motifs, as exemplified by directed evolution approaches,^270,274^ which might enable the development of tailor-made sortase enzymes that recognize the native C-termini of Ub and different Ubls.

##### 2.2.3.2 Ubl Topologies via Orthogonal Sortylation (Ubl-tools)

Recently, the identification of orthogonal sortase enzymes paved the way for the expansion of sortylation toward the generation of complex polymeric Ub/Ubl topologies. Orthogonal sortase variants have specificity for distinct and mutually orthogonal recognition motifs and are thereby suitable for iterative sortylation. Our group has identified the previously engineered sortases Srt2A (recognition motif LAXTG)^270^ and Srt5M (recognition motif LPXTG)^285^ as a bidirectionally orthogonal sortase pair that can be used for the iterative ligation of Ub and Ubl monomers.^286^ For this, bifunctional Ub and Ubl monomers were designed. They each present one of the two orthogonal sortase recognition motifs at their respective C-termini and bear a site-specifically introduced AzGGisoK handle; thus, they can serve as donor and acceptor moieties in orthogonal sortylation reactions. Iterative sortylation of these bifunctional Ub/Ubl variants using either Srt2A or Srt5M allowed the assembly of defined Ub/Ubl chains (Figure 9d). The versatility of this approach, which we dubbed Ubl-tools (Ubl-topologies via orthogonal sortylation), was demonstrated by the controlled generation of various homo- and heterotypic polyUb-POI conjugates. Importantly, as discussed above, through a small adaptation, sortylation can be made orthogonal toward endogenous ubiquitylation and therefore Ubl-tools can be used together with traditional ubiquitylation methods employing E1 and E2 enzymes. Combining Ubl-tools using the orthogonal sortase pair (Srt2A and Srt5M) with enzymatic ubiquitylation harnessing UBE1 and the K48-linkage-specific E2 enzyme CDC34 afforded a POI that was site-specifically modified with a K48-linked tetraUb chain (Figure 9d). Moreover, dual amber suppression within the same Ub monomer, bearing a C-terminus for Srt5M, allowed for the site-specific incorporation of two GGisoK moieties at K11 and K48. In a next step, Srt2A-mediated ligation enabled the facile generation of a defined branched tri-Ub chain that was subsequently charged onto GGisoK-bearing POI using Srt5M (Figure 9d). POIs linked site-specifically to defined types of Ub-chains may prove helpful in cryo-EM studies of the proteasome, elucidating the significance of K48-linked chains versus K11/K48 branched chains in the context of proteasomal degradation. The utility of Ubl-tools was further showcased by the generation of K63-diUb-SUMO2 hybrid chains that are involved in the DNA double strand break response (Figure 9d). Using Srt5M- and Srt2A-based sortylation in combination with enzymatic K63-diUb assembly, K63-linked diUbs were charged onto different lysine positions within SUMO2. Fluorescence anisotropy and NMR titration experiments revealed a previously unknown binding mode of K63-diUb-SUMO2 hybrid chains to Rap80, a BRCA1 adaptor protein involved in DNA repair.^286^

In conclusion, Ubl-tools is an elegant method for accessing complex and defined Ub/Ubl chains as well as defined polyUb/Ubl-POI conjugates in a controlled, easily applicable, and modular way. As described for sortylation, Ubl-tools could also be combined with the incorporation of ncAAs bearing photocrosslinker or chemical crosslinker moieties into one of the Ub/Ubl monomers. This would allow the identification of receptor and effector proteins that translate these complex Ub/Ubl patterns into specific biological outcomes. Similar to sortylation, as a potential disadvantage, Ubl-tools-generated Ub/Ubl chains also carry two point mutations in the linker regions between the monomers and therefore careful examination is needed to evaluate if these modifications may hamper important interactions. Furthermore, in its current form, Ubl-tools can only be applied *in vitro* and in an iterative way; one-pot or *in cellulo* approaches would not only need mutually orthogonal sortase recognition motifs installed at the Ub/Ubl C-termini but also require orthogonal nucleophilic acceptors.

##### 2.2.3.3 Asparaginyl Endopeptidase-Mediated Ubiquitylation

A second chemoenzymatic strategy for site-specific ubiquitylation relies on the use of an asparaginyl endopeptidase (AEP) from the plant *Oldenlandia affinis* (*Oa*AEP1, Figure 9e and f). AEP enzymes, first identified in plants but also found in animals including mammals, are cysteine proteases that selectively target peptide bonds C-terminal of asparagine or aspartate residues.^287,288^ While most AEPs catalyze the proteolytic cleavage of their targets, several members of the protein family (such as the ligating AEP1 enzymes butelase1 and *Oa*AEP1) were shown to possess transpeptidation activity and are involved in cyclotide biosynthesis.^289^ As chemical biology tools, both butelase1 and *Oa*AEP1 were successfully leveraged for *in vitro* macrocyclization reactions as well as N- and C-terminal protein labeling.^288−290^*Oa*AEP1 recognizes the tripeptidic motif NGL and cleaves the peptide bond between asparagine and glycine, resulting in a thioester intermediate between the target asparagine carboxylate and the *Oa*AEP1 active site cysteine thiolate. While *Oa*AEP1 shows quite strict sequence requirements for its recognition site and NGL represents an ideal motif, it is quite promiscuous for the nucleophile that attacks the thioester intermediate, with GL and GV dipeptides presenting efficient nucleophile acceptors.^290^ If present at high enough molar excess (typically ca. 1000 equiv compared to protein bearing NGL motif), various other primary amines were shown to be accepted as nucleophile acceptors by *Oa*AEP1.^291^ In an approach mirroring sortylation, our group recently showed that GGisoK can also act as an acceptor nucleophile for an engineered version of *Oa*AEP1 with enhanced catalytic efficiency (bearing the gatekeeper mutation C247A^292^) and can be employed for site-specific ubiquitylation and SUMOylation.^293^ The enzyme was shown to mediate the ligation between a donor Ub bearing a C-terminal *Oa*AEP1 recognition motif and the primary amine of GGisoK that was site-specifically incorporated into the acceptor protein by GCE and Staudinger reduction (Figure 9f). Installing the NGL recognition motif into the donor Ub requires the introduction of two point mutations, altering the C-terminal sequence from LRLRGG to LRLNGL. Nevertheless, the product of *Oa*AEP1-mediated ubiquitylation only differs from the native modification in position R74N because G76 is restored by the use of GGisoK as acceptor (Figure 9e). The resulting NGG motif is no longer recognized by *Oa*AEP1, which makes the transpeptidation irreversible. Moreover, Ub-POI conjugates displaying this single point mutation are susceptible to DUB cleavage, rendering *Oa*AEP1-mediated ubiquitylation a valuable complementary approach to sortylation. The applicability of *Oa*AEP1-mediated ubiquitylation was demonstrated by the generation of K48-linked diUb, site-selective ubiquitylation of histone H3 in three different positions, and the expansion of the approach to SUMOylation of Ub at position K48. The reaction is fast at neutral pH and works under mild aqueous conditions, requiring only small amounts of enzyme (0.01–0.04 mol equivalents). A potential drawback of this method is its limitation to *in vitro* applications, since *Oa*AEP1 needs to be activated at acidic pH and is therefore not suitable for *in vivo* experiments. In this regard, the recent identification of a truncated yet functional version of *Oa*AEP1 C247A that bypasses the acidic activation step and allows OaAEP1-mediated peptide cyclization in living *E. coli*, may become interesting.^294^ Nevertheless, the minimal recognition motif (NGL) and the promiscuity regarding nucleophile acceptors of *Oa*AEP1 also constitute double-edged swords: depending on the target proteins, unwanted side products may arise from attack on off-target asparagine residues in unstructured regions such as loop structures, especially if they are followed by small and hydrophobic amino acids. Furthermore, N-terminal mislabeling may also constitute an unwanted side reaction.

Sortylation and *Oa*AEP1-mediated generation of Ub/Ubl-POI conjugates constitute mutual chemoenzymatic approaches for making defined Ub/Ubl-POI conjugates. Furthermore, both approaches can be combined with endogenous ubiquitylation, employing linkage-specific E1 and E2 enzymes, allowing the generation of complex Ub/Ubl chains and polyUb/Ubl-POI conjugates where DUB-resistant and DUB-susceptible linkages can be placed at user-defined positions. Directed evolution approaches could further open up the possibility to create transpeptidases that target native Ub and Ubl C-termini or to discover novel orthogonal transpeptidases to generate other Ub/Ubl topologies with ease. In addition, combining the “ligation handle” presented by GGisoK with a crosslinking moiety within one ncAA could expand possible applications for chemoenzymatic site-specific ubiquitylation in the future.

## 3 PTMs Targeting Ubls: The Next Layer of Complexity

**Figure 10:**
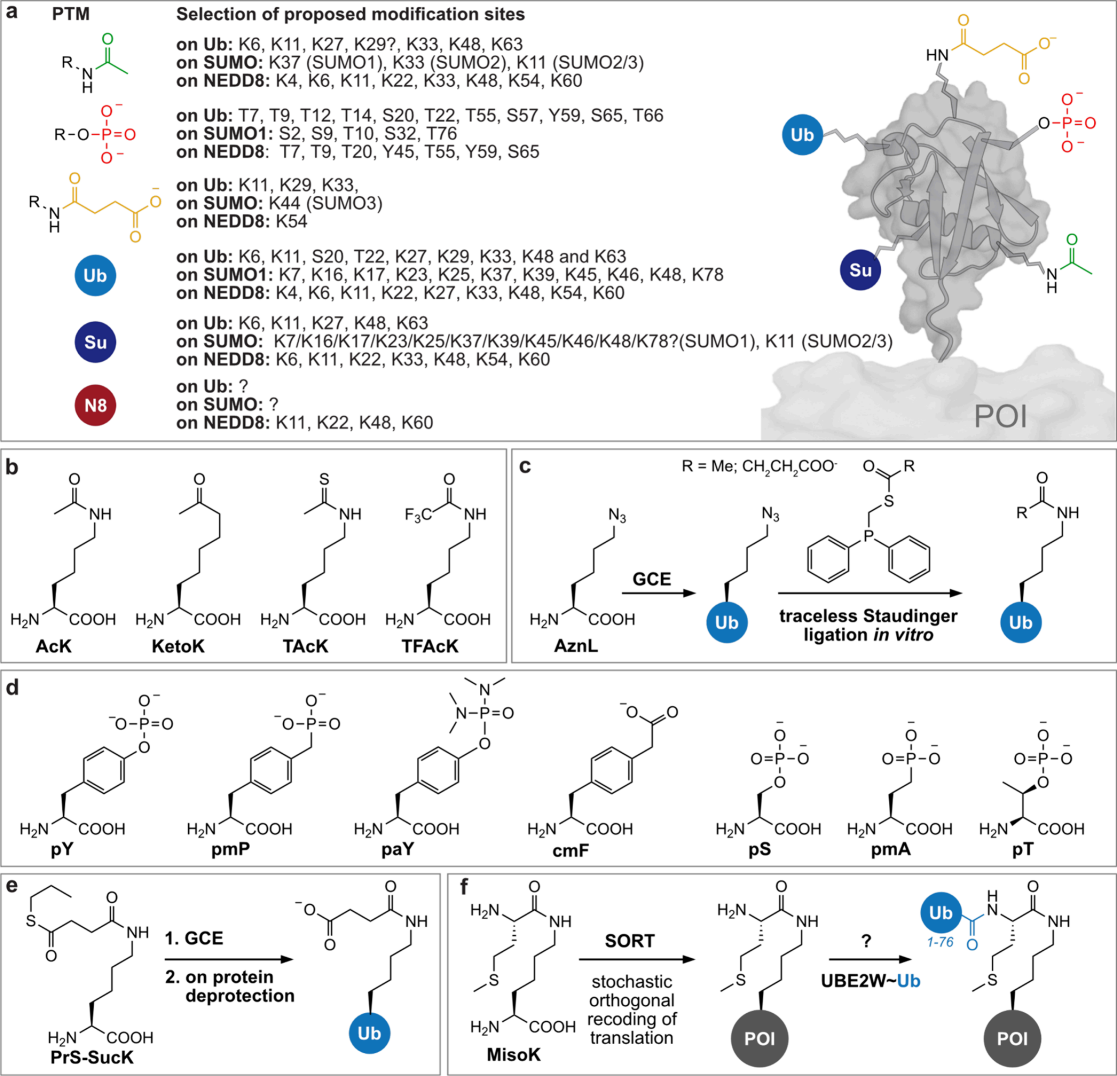
Interplay of ubiquitylation with other post-translational modifications. (a) Schematic representation of a post-translationally modified Ub linked to a POI and a list of reported and proposed PTMs of Ub and Ubls. Ub PTMs such as deamidation, ADP-ribosylation, and arginine phosphoribosylation are not shown. (b) Structures of AcK and AcK mimics such as KetoK, TAcK, and TFAcK. (c) Site-specific incorporation of AznL allows the installation of lysine acylations via traceless Staudinger ligation with phosphinothioesters (R = CH_3_, CH_2_CH_2_COO^–^) in 30−40% yield. (d) Structures of ncAAs that mimic tyrosine, serine, and threonine phosphorylation. pY can be mimicked by using pmP, paY or cmF. The phosphoramidate-bearing ncAA paY can be converted on a protein to pY via acidic treatment. pS, pT, and the nonhydrolyzable analogue pmA can be site-specifically incorporated into proteins via GCE. (e) PrS-SucK can be site-specifically incorporated into POIs by GCE. On-protein thioester hydrolysis affords succinylated POIs. (f) Stochastic orthogonal recoding of translation (SORT) facilitates proteome-wide incorporation of MisoK in response to a fraction of lysine codons. Proteomic studies revealed the ubiquitylation of MisoK, and downstream biochemical studies identified UBE2W as the E2 enzyme responsible for the ubiquitylation of aminoacylated lysines.

Being a protein-based PTM, Ub can be post-translationally modified itself, giving rise to an intricate metalayer of distinct modification patterns. As discussed above, the N-terminus as well as all seven lysine residues of Ub can act as acceptors for further ubiquitylation events, which leads to the formation of a plethora of sophisticated Ub topologies including heterologous, branched, and mixed Ub chains. Beyond the attachment of protein-based PTMs, small chemical modifications such as acetylation,^295^ phosphorylation,^296^ deamidation,^297^ ADP-ribosylation,^298^ and arginine phosphoribosylation^299^ of specific residues within Ub and other Ubls add yet another level of complexity to the Ub code. While some Ub/Ubl modifications and their regulatory effects have been investigated and the topic of previous reviews,^23,300^ PTM databases such as PhosphoSitePlus (http://www.phosphosite.org/)^301^ and CPLM 4.0 (http://cplm.biocuckoo.cn/)^302^ suggest the existence of many additional uncharacterized Ub/Ubl modifications (Figure 10a). In the following sections we will highlight how GCE-based approaches have helped decipher the role of site-specific small-molecule PTMs in Ub and Ubls and could equip future research with ideal tools to unveil further intricacies of Ub-PTM crosstalk (Figure 10).

### 3.1 Acetylation

More than half a century ago, modification of amine moieties in proteins by acetyl groups was discovered and characterized as a PTM.^303^ Protein acetylation, most famously studied in the context of transcriptional regulation orchestrated by histone acetylation,^304,305^ consists of the covalent attachment of an acetyl group to the protein N-terminus or to the *N*ε-amino group of a lysine residue, leading to a change in net charge from positive (+1) to neutral at physiological pH. Over the past decade, progress in proteomics has made it possible to characterize the cellular acetylome and discover many off-histone acetylation sites, including the acetylation of Ub and Ubls.^23,306,301,302,304^ With the possible exception of K29, acetylation of all six remaining lysine residues of Ub has been reported so far,^23,302,307^ which has important implications for Ub chain formation.^295^ In order to study the effects of defined acetylation events, ncAAs such as AcK (*N*ε-acetyl-l-lysine)^79^ and nonhydrolyzable derivates thereof like KetoK ((S)-2-amino-8-oxononanoic acid),^308^ TAcK (*N*ε-thioacetyl-l-lysine),^309^ and TFAcK (*N*ε-trifluoroacetyl-l-lysine)^310^ have been site-specifically incorporated into proteins via GCE (Figure 10b).

GCE approaches for site-specific Ub acetylation have so far been mainly used for *in vitro* studies, while *in cellulo* studies often still rely on more traditional and simple lysine to glutamine mutations to mimic AcK.^311^ AcK was one of the first amino acids to be site-specifically incorporated into target proteins using *Mb*- or *Mm*PylRS-derived variants.^79,312,313^ To improve the initially mediocre incorporation efficiencies of early AcKRS variants, a variety of engineering and evolution efforts have been undertaken^148^ to introduce mutations outside of the amino acid binding pocket^114^ as well as within the tRNA.^314^ Site-specific incorporation of AcK at positions K6 and K48 in Ub have shown that acetylation hampers Ub chain formation. *In vitro* chain assembly assays with wt Ub and acetylated variants in combination with a panel of E2 enzymes elucidated that Ub(K6AcK) and Ub(K48AcK) inhibited polyUb chain formation via K11, K48, and K63, presumably via inhibition of E2 access. For example, the interaction site of UEV1A (ubiquitin-conjugating enzyme E2 variant 1A), an E2 enzyme that promotes K63-linked polyubiquitylation, is close to K6 and K48 of the acceptor Ub, leading to decreased activity upon acetylation of one of these lysine residues. Interestingly, both acetylated variants were still competent donors for histone H2B(K120) monoubiquitylation by the E2 enzyme RAD6.^295^ A recent study by Shaw and co-workers optimized AcK incorporation in order to produce all possible Ub(AcK) variants in *E. coli* in good yields and analyzed their effect on two different E3 ligases in conjunction with the corresponding E2 enzymes.^315^ Surprisingly, the HECT-like E3 ligase IpaH3 and the RING E3 ligase RNF8, which are known to promote K48- and K63-linked polyubiquitylation, respectively, were still able to assemble polyUb chains *in vitro* even when the target lysines K48 and/or K63 were blocked by acetylation. While this observation implies a change in specificity of the conjugating machinery with regard to the generated linkage type upon blockage of the preferred linkage site by acetylation, the proposed effect was not studied in further detail. The structural and functional consequences of Ub acetylation were investigated more thoroughly in a recent study by Scheffner, Kovermann, Marx, and co-workers that was also based on site-specific incorporation of AcK at all seven possible sites within Ub.^316^ It was shown that Ub acetylations at positions K6, K11, K27, and K48 impair autoubiquitylation for several E3 ligases. For the K48-specific HECT E3 ligase E6AP in combination with Ub(K48AcK), this could be explained by the specificity of the E3. Yet, similar effects were also observed for unspecific E3s as well as for E6AP in combination with other Ub(AcK) variants. NMR studies revealed that in addition to loss of the positive charge, lysine acetylations induce, in a position-specific manner, subtle structural changes within Ub that may affect protein–protein interactions. This effect is especially pronounced for position K11, where acetylation leads to an altered I44 patch resulting in reduced binding to the UBA domain of ubiquilin-2. Interestingly, this was not the case for Ub(K11Q), which behaved like unmodified Ub, highlighting that the commonly used lysine to glutamine mutation cannot fully mimic acetylation in all positions. Furthermore, it was shown that for positions K11, K27, K29, K33, and K63, acetylation has a similar effect on the Ub structure as ubiquitylation, potentially leading to comparable interaction properties of acetylated Ub and diUb. Affinity enrichment mass spectrometry experiments with unmodified Ub and all seven AcK-bearing variants in HEK293T lysates identified partly overlapping but nonetheless distinct interactomes for the different acetylation sites.^316^ Further insights into the effects of Ub acetylation were provided in a study by Lacoursiere and Shaw that investigated the kinetics of Ub∼E2 conjugate formation. For this, acetylated Ub variants were labeled with donor fluorophores, and the E2 enzyme UBE2D1 was labeled with an acceptor fluorophore. E1-mediated formation of the corresponding Ub∼UBE2D1 conjugates was followed via FRET (Förster resonance energy transfer). Site-specific incorporation of AcK into Ub revealed a correlation between the position of Ub acetylation and UBA1 catalytic activity, providing evidence that acetylation of Ub can modulate ubiquitylation pathways at early checkpoints.^317^ In conclusion, the incorporation of AcK into Ub with GCE has proven very valuable for studying the effects of Ub acetylation *in vitro*. The use of AcK in living eukaryotic cells is, however, limited due to the presence of deacetylases that catalyze the rapid hydrolysis of this PTM. This obstacle may potentially be overcome by the use of nonhydrolyzable AcK mimics, such as KetoK, TFAcK, and TAcK (Figure 10b). KetoK (Figure 10b) was designed as an ncAA bearing a site-specific reaction handle for bioorthogonal labeling with hydrazides and alkoxyamines but was also envisioned to serve as a stable AcK mimic.^308^ While it found use for oxime ligation-based protein labeling *in vitro*(205,318) and as an AcK mimic within p53(K120KetoK),^205^ KetoK has not found broad application as an AcK mimic on Ub, potentially also due to toxicity that has been observed for ketone-bearing ncAAs.^319^ The trifluoro acetyl-bearing TFAcK mimic and the thioacetyl-modified TAcK (Figure 10b) analog have been shown to be stable against hydrolysis by certain NAD^+^-dependent sirtuins and the bacterial deacylase CobB, respectively, as tested in *in vitro* assays.^309,310^ Still, so far, they have not been used to study Ub acetylation in eukaryotic cells.

An alternative method to direct incorporation of AcK or mimics thereof with GCE consists of a two-step approach to install various lysine acylations. The method, developed by Liu and co-workers, relies on the site-specific incorporation of AznL (azidonorleucine) via GCE followed by traceless Staudinger ligation with a phosphinothioester derivate (Figure 10c).^218^ This strategy has been used to acetylate and succinylate Ub at position K48 but suffers from its nonquantitative nature, since the undesired Staudinger reduction of the azide to the corresponding amine limits the yield of modified protein to roughly 30–40%, with wt Ub as side product. Hence, it is difficult, if not impossible to homogeneously purify the modified Ub variant. Moreover, the method is inherently limited to *in vitro* applications.

Beside Ub acetylation, SUMO acetylation has also been reported (Figure 10a).^301,302,306^ Pioneering studies by Müller and co-workers focused on the acetylation of lysine residues residing at the interaction interface between SUMO and SIMs (SUMO-interaction motifs) of SUMO-binding proteins. *In vitro* pull-down assays with SUMO1(K37AcK) and SUMO2(K33AcK), generated via site-specific incorporation of AcK, showed that the acetylated variants failed to associate with SIMs in SUMO-binding proteins such as the SUMO E3 ligase PIAS1, Daxx (death domain-associated protein 6), and PML (promyelocytic leukemia protein), while nonacetylated SUMO variants showed strong interaction. Surprisingly, the interaction between SUMO1 and the SUMO E3 ligase RanBP2 (Ran-binding protein 2) is independent of the SUMO1(K37) acetylation status, indicating that the positive charge of K37 is not required for binding to the SIM of RanBP2.^306^ Analogously to Ub, SUMO variants are able to form polymeric chains that are preferentially linked via K11. MS-based approaches showed that K11 can undergo acetylation, raising the question of how this PTM impacts the polymerization of SUMO.^320^ The Müller group used site-specific incorporation of AcK at position K11 within SUMO2 and revealed that SUMO chain formation is strongly inhibited by acetylation and that SUMO2(K11AcK) is able to terminate polySUMO chains. On a similar note, SUMO2(K11AcK) did not influence the monoSUMOylation of p53 in an *in vitro* SUMOylation assay in rabbit reticulocyte lysate but strongly reduced K11-linked SUMO chain formation on p53.^320^ Furthermore, *in vitro* deacetylation assays showed that SIRT1 displays deacetylation activity toward SUMO2(K11AcK) but does not affect acetylation at K33 and K35. In contrast, SIRT2 was not able to deacetylate SUMO(K11AcK). Subsequent *in vitro* and cell lysate assays demonstrated that SIRT1 can restore SUMO chain elongation by deacetylating SUMO2(K11AcK).^320^ Taken together, acetylation of Ub and SUMO serves as an additional regulatory layer of the Ub- and SUMO-system that modulates the catalytic activity of the writer and eraser machinery to influence Ubl modification as well as polymerization status and fine-tunes downstream protein–Ubl interactions. It should be noted that Ubl acetylation is not limited to SUMO. A recent study by Kurz and co-workers observed the acetylation of NEDD8 on K11, K22, K33, and K48 and investigated the acetylation of unanchored NEDD8 trimers in response to oxidative stress. It was shown that the inhibiting effect of the PTM on PARP1 (poly(ADP-ribose) polymerase 1) was reversed upon overexpression of deacetylases.^321^

### 3.2 Phosphorylation

Arguably the best studied PTM involved in almost every cellular process is protein phosphorylation catalyzed by kinases.^322^ Phosphorylation events can occur either via O-phosphorylation on serine, threonine, and tyrosine yielding phosphates or via less stable N-phosphorylation on lysine, arginine,^323^ and histidine^324^ resulting in phosphoramidates. Furthermore, aspartate phosphorylation via acylphosphates^325,326^ and cysteine phosphorylation via thiophosphate^327^ bonds have been described. Concerning the Ub system, MS-based approaches have identified O-phosphorylation sites spread over almost all serine, threonine, and tyrosine residues of Ub.^44,301^ In order to study the effects of phosphorylation, GCE enabled the incorporation of a vast panel of phosphorylated amino acids and derivates thereof, although the incorporation of ncAAs introducing these PTMs poses unique challenges: the negative charge of the phosphate group impairs cell permeability, renders phospho-ncAAs less suited substrates for the hydrophobic active site of PylRS variants, and may also potentially impair binding to EF-Tu. Furthermore, endogenous phosphatases will reverse phosphorylation and may directly act on phosphor-ncAAs and mimics thereof.^328^ Strategies for overcoming these limitations range from the use of engineered expression hosts and translation machineries to approaches for increasing the cellular availability of phosho-ncAAs or masking their negative charge to use of phosphatase-resistant analogs and were recently reviewed in detail.^329,148^

#### 3.2.1 Tyrosine Phosphorylation

The site-specific incorporation of pY (phosphotyrosine, Figure 10d) into target proteins was achieved by Söll and co-workers via direct incorporation of pY by an engineered *Mj*TyrRS/tRNA pair employing an optimized EF-Tu variant in an *E. coli* strain lacking five phosphatases.^328^ Shortly after, Schultz, Wang, and co-workers used a dipeptide of pY (KpY) to promote active uptake in *E. coli* for the incorporation of pY by employing an engineered *Mj*TyrRS/tRNA pair. As MS analysis revealed 90% phosphate hydrolysis despite the addition of phosphatase inhibitors during protein expression, the same dipeptide strategy was successfully employed for incorporating the nonhydrolyzable pY analog Pmp (4-phosphomethyl-l-phenylalanine, Figure 10d).^156^ Wang and co-workers successfully incorporated a phosphoramidate derivative of pY (paY, Figure 10d) with an engineered *Mm*PylRS/tRNA pair followed by *in vitro* acidic deprotection to afford pY-modified proteins.^157^ In addition, the nonhydrolyzable pY mimic cmF (p-carboxymethyl-l-phenylalanine, Figure 10d) was successfully incorporated using a *Mj*TyrRS variant.^156,330^ The impact of tyrosine phosphorylation on Ub conformation and function is of great interest since this PTM has so far been observed exclusively in cancerous tissue.^331^ There is only one tyrosine residue, Y59, within the Ub sequence. Mutating Y59 to alanine revealed that a hydrogen bond between the Y59 side chain and the E51 backbone amide (Y59-E51 loop) is crucial for Cdc34-mediated formation of K48-linked Ub chains.^332^ Incorporation of the phosphoramidate derivative paY at position 59 followed by acidic treatment to reveal pY enabled NMR studies on the structural effects of tyrosine phosphorylation on Ub. Compared to wt Ub, Ub(Y59pY) displays dramatic changes in the conformation of the Y59-E51 loop that lead to significantly decreased charging onto the E2 enzyme UBE2D3, as demonstrated in E1/E2 autoubiquitylation assays.^157^ Even though this study gives a first insight into Y59 phosphorylation, its functional interplay with other components of the ubiquitin system such as E3 ligases and readers remains elusive and warrants further studies. The toolbox for studying tyrosine phosphorylation of Ub was expanded recently by the development of specific binders for folded Ub(Y59pY). To generate Ub(Y59pY) required for the engineering and validation of the binders, paY was incorporated into Ub via GCE followed by acidic deprotection to afford pY.^333^ The evolved Ub(Y59pY) binder may prove beneficial to assign biological function, elucidate the upstream kinases and study signaling regulations of Ub tyrosine phosphorylation.

#### 3.2.2 Serine and Threonine Phosphorylation

In contrast to the still enigmatic biological role of Ub tyrosine phosphorylation, phosphorylations of serine residues (pS, Figure 10d) within Ub have been studied in more detail. By combining the orthogonal pS-tRNA synthetase (SepRS) from *Mmp* with a cysteinyl-tRNA from *Mj* (with engineered anticodon recoding the amber codon) and engineering EF-Tu, Söll and co-workers first showed the site-specific incorporation of pS (*O*-phosphoserine, Figure 10d) into target proteins in *E. coli*.^85^ Additional optimizations, such as partial TAG codon reassignment as well as depletion of RF1 and several phosphatases, increased yields of the phosphorylated protein.^334^ Further genetic and metabolic engineering led to an *E. coli* strain showing elevated levels of phosphorylated amino acids.^335^ The Chin group boosted pS-incorporation by evolving the nucleic acid sequence surrounding the anticodon in *Mj*tRNA as well as the tRNA binding region within SepRS and showed efficient the incorporation of the nonhydrolyzable pS analog pmA (*N*-(phosphomethyl)-l-alanine, Figure 10d).^86^ Further amendments by engineering an EF-1α derivative as well as using an eRF1 mutant combined with manipulations of pS biosynthesis allowed the incorporation of pS and its nonhydrolyzable phosphonate analog pmA into proteins expressed in mammalian cells.^88^ In parallel, deep sequencing as well as inserting a biosynthetic pathway for efficient production of phosphothreonine (pT, Figure 10d) in *E. coli* allowed the evolution of a tRNA synthetase that directs the site-specific incorporation of pT in bacteria.^87^

The most prominently studied example of Ub phosphorylation is represented by the phosphorylation of serine 65 within Ub and its role in Parkinson’s disease. In brief, PINK1 (serine/threonine-protein kinase 1) and the E3-ligase Parkin play a crucial role in familiar parkinsonism by depleting damaged mitochondria via the induction of mitophagy. The connection between these key players has long been elusive.^336^ Groundbreaking investigations uncovered that Ub is a substrate of PINK1,^337^ while subsequent structural studies elucidated the mechanism of PINK1-mediated Ub phosphorylation^338^ and how phosphorylated Ub activates Parkin.^339^ The current model suggests that PINK1 stabilization leads to S65 phosphorylation of Ub that is attached to proteins on the outer mitochondrial membrane (OMM). Due to its high affinity to Ub(S65pS), cytosolic autoinhibited Parkin gets translocated to the OMM and is activated through the binding of Ub(S65pS). The UBL domain of Parkin is then phosphorylated by PINK1, which leads to downstream effects resulting in mitophagy.^336^

Using the above-mentioned optimized approach for pS incorporation, Chin and co-workers generated Ub(S65pS), which was used in benchmarking experiments to prove its structural and functional similarity to enzymatically derived Ub(S65pS). Both enzymatically- and GCE-derived Ub(S65pS) performed identically in E2-discharging and in Parkin activation assays.^86^ Furthermore, GCE allowed the generation of hitherto inaccessible phosphorylated Ub variants, such as Ub(S7pS), Ub(S12pS), Ub(S20pS) and Ub(S57pS).^340^ Biochemical studies revealed that Ub(S12pS) was able to partially activate Parkin, although to a lower extent than Ub(S65pS), while Ub(S20pS) completely failed to activate Parkin. Most surprisingly, Ub(S57pS) was shown to hyperactivate Parkin, possibly indicating a PINK1-independent route for Parkin activation.^340,341^ This constitutes an especially interesting finding, considering that phosphorylation of S57 is the most abundant phosphorylation observed for Ub.^342^ In a rigorous combinatorial study, GCE in conjunction with enzymatic assembly facilitated the generation of 20 different isomeric phospho-diUbs with different sites of isopeptide linkages and/or phosphorylations at S20, S57, or S65.^343^ This panel of phospho-diUbs not only allowed large scale DUB profiling with 31 different DUBs, unraveling the regulatory potential of Ub phosphorylation in activating and repressing DUB activity, but also revealed that phosphorylation of Ub at position S20 regulates the specificity of the E3 ligase UBE3C.^343^ Systematic profiling of small-molecule PTMs combined with differently linked Ub/Ubl chains, as exemplified in this study, may represent a promising strategy to further dissect the additional regulatory layer encoded in small-molecule PTMs attached to Ubls. It should be noted that Ub and diUbs modified by serine phosphorylation or a stable mimic thereof were also accessed by total chemical synthesis in recent years.^344,345^ Although labor-intensive and typically only suited for specialized laboratories with expertise in chemical protein synthesis, such approaches can be advantageous for introduction of multiple pSer modifications and for the generation of diUb conjugates bearing asymmetric phosphorylation patterns.

With regard to other Ubls, phosphorylation was also identified as a PTM on S2 of SUMO1 and SUMO3^346^ as well as on T76 of SUMO1, where it was reported to increase the half-life of SUMO1.^347^ Interestingly, Akt, the kinase responsible for the phosphorylation of SUMO1 T76, is also regulated by SUMOylation, which again highlights the complexity and intricate interplay of the Ubl code.^347^

NEDD8 gets phosphorylated at S65, analogously to the well characterized Ub S65 phosphorylation, and has been shown to also allosterically activate parkin.^348^ Site-specific incorporation of pS and the nonhydrolyzable analogue pmA served as the basis for *in vitro* studies on homogeneously phosphorylated NEDD8 and Ub. NMR experiments revealed that phosphorylation at S65 causes similar structural changes for Ub and NEDD8, and that pmA serves as good mimic of pS. Despite similar structural features of Ub(S65pmA) and NEDD8(S65pmA), they showed limited overlap in their interactomes in HEK293T and SH-SY5Y cell lysates as determined by affinity enrichment mass spectrometry. Importantly, their interactomes are also distinct from those of unmodified Ub and NEDD8. Strong enrichment of Hsp70 family proteins by phosphorylated NEDD8 but not by phosphorylated Ub led to the discovery that NEDD8 phosphorylation increases the affinity of NEDD8 to Hsp70 as well as the subsequent stimulation of Hsp70, which was further demonstrated by *in vitro* ATPase assays.^348^ These results showcase how phosphorylated NEDD8 can also act as a noncovalent modulator, as exemplified by its involvement in stress response pathways.

### 3.3 Succinylation

Although succinylation of Ub remains more elusive than acetylation and phosphorylation, the PTM has been reported to modify several residues of Ub, including K48 and K33.^349^ In comparison to acetylation, succinylation increases the size of the lysine side chain more considerably and reverses its charge from +1 to −1 under physiological conditions, likely impacting structure, function and interactomes of target partners. As mentioned above, a two-step approach based on Staudinger ligation has been used to generate succinylated Ub, but in only 30–40% yield and with wild-type lysine-bearing Ub as the main byproduct, making it impossible to obtain homogeneous SucK-modified Ub as needed for functional biochemical studies.^218^ Our group recently developed a more suitable approach to directly incorporate this PTM for the facile and homogeneous generation of site-specifically succinylated proteins.^152^ Masking the negatively charged side chain of SucK (succinyllysine) with an *S*-propyl thioester (PrS-SucK) moiety allowed for the incorporation of PrS-SucK by *Mb*PylRS and *Ma*PylRS variants dubbed ThioRS via GCE (Figure 10e). Subsequent on-protein hydrolysis of the thioester moiety by either β-mercaptoethanol treatment or incubation at slightly elevated pH (pH 8–9) restored the negatively charged lysine acylation. In the context of the Ub system, the method was employed to study the effects of Ub succinylation at position K33 on the interaction with the K11-specific DUB cezanne. Residue E157 of cezanne forms a salt bridge with K33 of the proximal Ub in K11-linked Ub chains. Comparing cezanne-induced hydrolysis rates of K11-linked wt diUb versus the rates of a K11 diUb bearing the K33SucK modification in the proximal Ub showed the impaired hydrolysis of the succinylated variant, likely caused by electrostatic repulsion between cezanne E157 and the succinylated lysine in Ub.^152^ This indicates a regulatory potential of succinylation in repressing or potentially also increasing DUB activity, as has been shown for phosphorylation.^343^

### 3.4 Aminoacylation

Reversible aminoacylation of lysine with all 20 proteinogenic amino acids has been characterized and reported recently as a novel PTM, which is presumably installed on proteins by aminoacyl tRNA synthetases and reversed by deacylases.^350^ Based on this hypothesis, a recent study by Lin and co-workers set out to investigate this PTM in the context of ubiquitylation, raising the question of whether aminoacylated lysine residues in target proteins can undergo ubiquitylation and what potential consequences such a secondary PTM might entail.^351^ Using *Mb*PylRS and an engineered tRNA variant that allows incorporation of the ncAA methionyl-lysine (MisoK, Figure 10f) in response to lysine sense codons (stochastic orthogonal recoding of translation, SORT^352^), lysine residues all over the proteome of HEK293T cells were in low-frequency replaced by MisoK. Subsequent trypsin digestion and proteomic analysis identified several sites that displayed a characteristic diglycine modification on MisoK, indicating potential ubiquitylation of these sites. In-depth proteomic analysis of unmanipulated cells and re-evaluation of a previously published UbiSite data set^353^ provided further evidence that hinted at the possible ubiquitylation of lysine residues aminoacylated with all 20 proteinogenic amino acids.^351^ The authors conclude that ubiquitylation of aminoacylated lysine residues may lead to accelerated proteasomal protein degradation, complementing canonical lysine ubiquitylation. It was proposed that the E2 enzyme UBE2W is responsible for ubiquitylation of aminoacylated lysines, as it was discharged by aminoacylated lysine in *in vitro* assays and, furthermore, a positive correlation between UBE2W expression in HEK293T cells and the frequency of aminoacylated lysine ubiquitylation was observed.^351^ While *in vitro* biochemical data on UBE2W-mediated ubiquitylation of MisoK-modified (or more generally aa-isoK-modified proteins) is missing and knowledge on this secondary PTM is limited so far, necessitating further studies and proof, it represents an interesting example for potential noncanonical ubiquitylation events and crosstalk between ubiquitylation and other PTMs.

In conclusion, the studies of small chemical PTMs on Ub such as acetylation and phosphorylation aided by GCE have so far granted profound insights into this new metalevel of regulation. This knowledge must now be expanded systematically both toward the crosstalk between these PTMs and their impact on the remaining Ubls. A promising possibility relies in the discovery of mutually orthogonal aaRS/tRNA pairs, which would in theory allow the simultaneous incorporation of a small chemical PTM or an analogue thereof together with a conjugation handle at distinct positions within one Ub molecule to allow the formation of Ub architectures with defined linkages and a tailor-made PTM status. Taken together, we are just at the beginning of a new understanding how the ubiquitin code is regulated by these modifications and how they impact (1) the structure of Ub itself, (2) the regulation of the conjugation and deconjugation machineries, and (3) the downstream events triggered by readers of the ubiquitin code.

## 4 GCE-Based Ub/Ubl Probes to Investigate the Ub/Ubl System

The intricacies and complexities of the Ub/Ubl system rely on a multitude of transient PPIs that allow dynamic control of cellular processes. These interactions are typically brief and weak but critical for the selective and rapid modification of substrate proteins.^6^ They ensure flexibility and precision in cellular regulation and allow for rapid assembly and disassembly of complexes between E2 and E3 enzymes, between E3 enzymes and their target proteins, and between ubiquitylated proteins and DUBs; therefore, they are critical for responding swiftly to cellular needs and environmental changes. Furthermore, low-affinity binding also contributes to the specificity of the Ub/Ubl system, preventing prolonged or inappropriate interactions that could disrupt cellular processes and allowing for modulation and adaptation in a timely manner. To gain a deeper understanding of the Ub/Ubl system, it is indispensable to study when and where these interactions occur. Due to their fleeting and weak nature, however, this poses a considerable challenge. Recent advances in chemical biology and biophysical techniques, such as affinity-based probes and activity-based profiling combined with proteomics, imaging, and structural biology, have enabled researchers to capture, stabilize and analyze these transient interactions effectively.^354−357^ The possibility to site-specifically introduce reactive groups such as photocrosslinkers, chemical crosslinkers, or bioorthogonal handles into components of the Ub/Ubl system via GCE opens up the exciting possibility to map transient PPIs and capture low-affinity protein complexes of the Ub/Ubl system within living cells. Such tools complement the vast and still growing palette of synthetic and semisynthetic Ub/Ubl activity-based probes (ABPs),^50,357^ and benefit from their potential of being directly generated in living cells, bypassing the crucial hurdle of cell-permeability.^358,359^

### 4.1 Genetically Encoding Crosslinking Moieties to Study PPIs within the Ub/Ubl System

**Figure 11:**
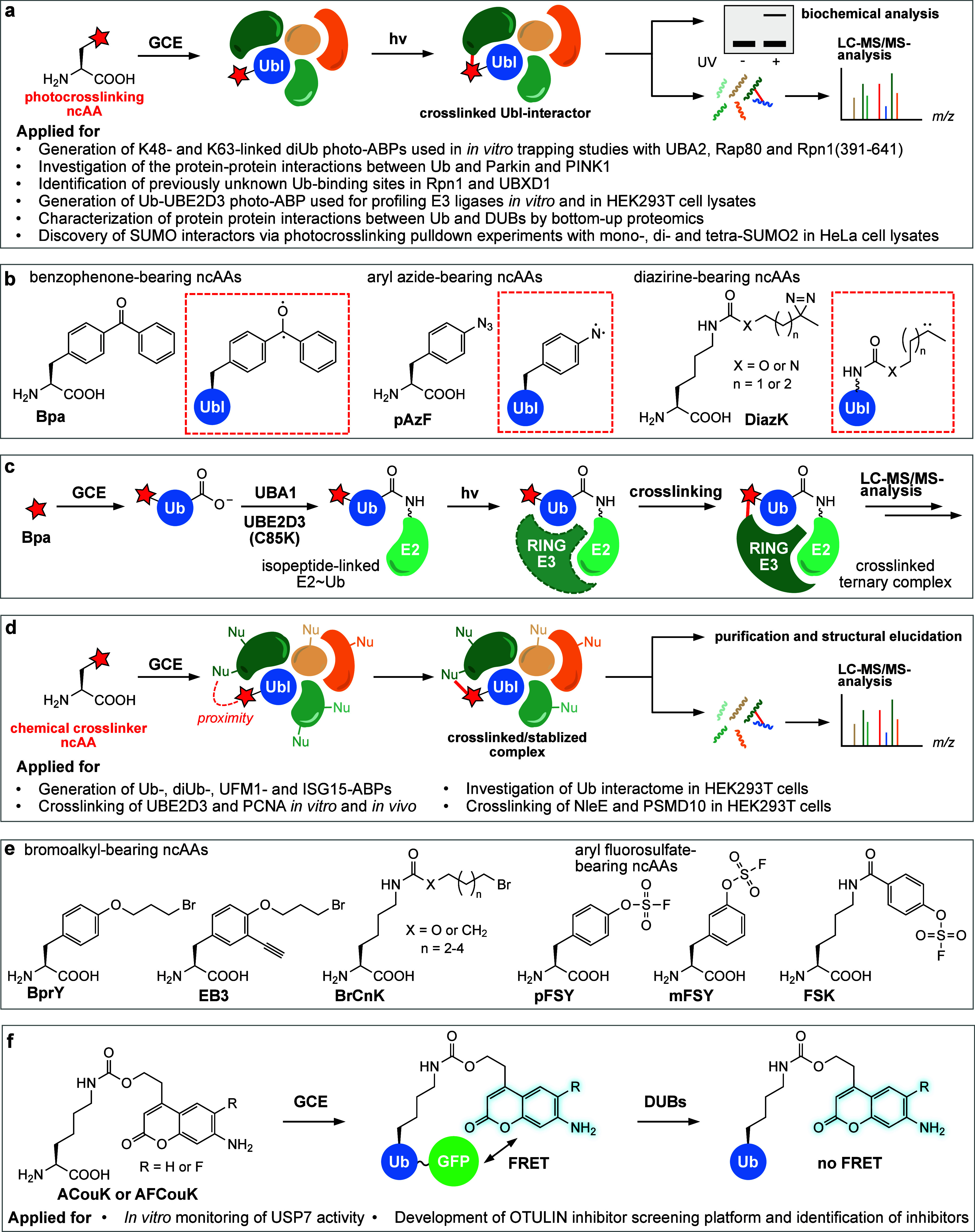
GCE-based Ub/Ubl probes to investigate the Ub/Ubl system. (a) Schematic representation and applications of GCE-based photocrosslinking Ub probes. GCE facilitates the site-specific incorporation of ncAAs bearing a photocrosslinking moiety. Irradiation with UV light leads to the formation of a highly reactive species that covalently crosslinks to molecules in proximity of the ncAA, *e.g.*, proteins that engage in PPIs with Ub. LC-MS/MS and biochemical assays further elucidate the interactome of the Ub probe. (b) Structures of photocrosslinking ncAAs Bpa, pAzF, and DiazK and their respective highly reactive species on amber-suppressed proteins irradiated with UV light. (c) The E1 enzyme UBA1 charges Bpa-bearing Ub onto UBE2D3(C85K), yielding a stable isopeptide bond instead of the native Ub∼E2 thioester. Photocrosslinking and subsequent LC-MS/MS analysis identify interacting RING E3 ligases. (d) Schematic display and applications of GCE-based chemical crosslinking Ub probes. GCE facilitates the site-specific incorporation of chemical crosslinker ncAAs bearing an electrophilic warhead that only reacts with nucleophiles in immediate proximity. LC-MS/MS and biochemical assays further elucidate the interactome of the Ub probe. (e) Structures of chemical crosslinker-bearing ncAAs that display either a bromoalkane or an aryl fluorosulfonate moiety. (f) GCE facilitates the incorporation of ACouK and AFCouK into a Ub-GFP fusion protein that serves as a FRET probe to monitor DUB activity. Upon DUB-mediated cleavage of GFP, loss of FRET can be observed.

ncAAs bearing a photocrosslinker moiety in their side chain are stable under physiological conditions but form highly reactive species upon UV light treatment and make covalent bonds with molecules in their vicinity. Their genetic encoding thereby provides—coupled with biochemical assays and advanced proteomics methods—a useful tool for mapping dynamic and weak protein interactions in living cells with spatial and temporal control (Figure 11a). Furthermore, such approaches may help define the structure and topology of protein complexes both *in vitro* and *in cellulo*. The repertoire of photocrosslinking ncAAs typically features benzophenone, diazirine, or aryl azide moieties that are all activated with UV light and form reactive ketyl radical, nitrene, or carbene intermediates, respectively, that covalently crosslink to neighboring molecules (Figure 11b).^138,283^ While the photocrosslinking ncAA Bpa (*p*-benzoyl-l-phenylalanine, Figure 11b) was already used in SPPS-based applications in the mid 1980s,^360^ its site-specific incorporation into proteins via GCE by an evolved *Mj*TyrRS synthetase variant in *E. coli* ca. 15 years later^361^ laid the foundation for the use of Bpa as a tool to study protein–protein interactions in living cells.

Not surprisingly, site-specific incorporation of photocrosslinker-bearing ncAAs has also found application as a tool for studying interactions between Ub/Ubls and their respective reader, writer, and eraser enzymes. The effect of Bpa incorporation in Ub was investigated in a study that focused on characterizing the properties of the resulting photoaffinity probes in a series of *in vitro* experiments.^362^ Incorporation of Bpa into different positions of Ub and subsequent *in vitro* photocrosslinking studies with the E2 enzyme UBA2 allowed the identification of hot spot positions within Ub showing enhanced crosslinking reactivity. Enzymatically prepared K48- and K63-linked diUbs bearing Bpa at position T9 or K48 in the proximal Ub or position T9 in the distal Ub were successful in capturing the respective binding partners (UBA2 or Rap80 tUIM, respectively). Two other studies used site-specific Bpa incorporation for the elucidation of PPIs between Ub and Parkin^363^ as well as Ub and PINK1^364^ and showed that the Ub I44 patch is important for both of these PPIs. Furthermore, photocrosslinking studies with the proteasomal subunit Rpn1 and Bpa-bearing monoUb, as well as K11, K48, and K63-linked Bpa-bearing diUbs, provided insights into the interaction between these proteins and revealed a previously unknown Ub-binding site in Rpn1.^365^ Similarly, photocrosslinking Ub(F4Bpa) recently contributed to the identification of a novel Ub binding site in the AAA+ ATPase VCP/p97 adaptor protein UBXD1.^366^ Bpa-based photocrosslinking was also used in a recent elegant approach to design activity-based photocrosslinking probes targeting RING E3 ligases.^282^ With over 600 members, RING E3s constitute by far the largest class of E3 enzymes. As they show adaptor-like activity that can be subject to various regulatory mechanisms and do not rely on a catalytic nucleophile but instead catalyze direct transfer of Ub from E2∼Ub to a substrate, ABPs for measuring RING E3 activity are difficult to design and engineer. Virdee and co-workers engineered a Bpa-bearing E2-Ub ABP by leveraging the E1 enzyme UBA1 to charge Bpa-bearing Ub(Q31Bpa) onto a variant of the E2 enzyme UBE2D3 that had the active site cysteine replaced by a lysine in order to form a more stable isopeptide linkage between E2 and Ub instead of the labile endogenous thioester linkage (Figure 11c).^282^ Importantly, structural analysis had shown that the isopeptide linkage was an acceptable structural mimic of the native thioester.^367−369^ Furthermore, the Ub-E2-photoABP was decorated with an affinity tag to facilitate purification and to serve as a convenient reporter tag for immunoblot analysis. The photoABP was used to profile the activation of the E3 ligases RNF4 and c-Cbl *in vitro* and in HEK293T cell extracts, respectively. Moreover, a subset of 25 RING E3 ligases was trapped by the photoABP in HEK293T lysates, further demonstrating the versatility of the probe.^282^ In a follow-up study, the same probe was used to monitor E3 ligase RNF12 catalytic activity in normal and pathogenic contexts.^370^ Apart from efficiently trapping writer and reader enzymes with Bpa-modified Ub, a recently published study used Bpa-bearing Ub(T9Bpa) and crosslinking mass spectrometry for characterizing interactions between Ub and eukaryotic DUBs of five different families as well as three bacterial DUBs.^371^

In addition to the studies focused on Ub discussed above, photocrosslinking ncAAs were also employed for the characterization of SUMO-interacting proteins. Noncovalent interactions between SUMO and its interaction partners are mainly dependent on the binding of the SIM to a hydrophobic groove on the SUMO surface. As inherent to most interactions within the Ub/Ubl system, SUMO-SIM interactions are also characterized by low affinity binding. *In cellulo* photocrosslinking via GCE therefore represents a suitable tool to trap and map these transient interactions.

The Mootz group used site-specific incorporation of Bpa as well as pAzF (*p*-azidophenylalanine, Figure 11b) into various positions of the SUMO1 SIM-binding groove and showed that Bpa/AzF-bearing SUMO1 was still accepted by the SUMO conjugating and deconjugating machinery, although the incorporation of the ncAAs had slight functional effects on SUMO1.^372^ Subsequent photocrosslinking studies with the SIM-containing proteins PIASX and RanBP2 showed successful crosslinking. A comparison of SUMO1(R54Bpa) and SUMO1(R54AzF) revealed that incorporation of the larger hydrophobic side chain of Bpa led to more undesired self-crosslinking of the probe, likely caused by its hydrophobic character and therefore increased aggregation tendency.^372^ Nevertheless, Bpa was used in a follow-up study by the same group that focused on the identification of previously unknown SUMO binding proteins.^373^ For this, SUMO2(R50Bpa) as well as mimics of K11-linked diSUMO2 and tetraSUMO2 chains (expressed as linear ΔN11 fusions) displaying Bpa on their proximal SUMO moiety were used in photocrosslinking pulldown experiments in nuclear HeLa cell extracts. The majority of the >300 captured proteins had not been reported as SUMO binder candidates in previous proteomic studies, yet analysis with SIM prediction tools showed that 90% of the hits contained one or multiple *in silico* predicted SIMs, validating the photocrosslinking approach for finding new SUMO interactors.^373^

Many writer and eraser enzymes of the Ub/Ubl system contain active site cysteines to activate, conjugate, and ligate Ub/Ubl as well as to hydrolyze the isopeptide bond in Ub/Ubl chains and Ub/Ubl-POI conjugates. SPPS- or EPL-generated ABPs based on monoUb or diUb bearing either a C-terminal or an internal electrophilic warhead that covalently traps proximal cysteine residues within interacting proteins are proven tools to interrogate enzyme specificities and elucidate enzyme mechanisms.^355,357,374,375^ To enable the stabilization of otherwise transient protein complexes even in the crowded environment of a living cell, the genetic encoding of latent and finely tuned electrophilic functionalities that can react with cysteines and potentially also with other nucleophilic canonical amino acids when in sufficient proximity has been explored (Figure 11d). A palette of ncAAs bearing electrophilic functionalities have been incorporated site-specifically into target proteins and have been shown to covalently react with different proximal nucleophilic natural amino acids.^138,283,376^ Most prominent and useful examples include bromoalkyl-bearing ncAAs such as BprY, EB3, and BrCnK (Figure 11e) that make covalent bonds with proximal cysteine, aspartate, or glutamate residues within living cells,^140,141,377^ as well as ncAAs featuring aryl fluorosulfate electrophiles that can engage in sulfur(VI) fluoride exchange (SuFEx) reactions^378^ with lysine, histidine, or tyrosine such as pFSY, mFSY, and FSK (Figure 11d).^379,380,283^ In a recent study, BprY (*O*-(3-bromopropyl)-tyrosine, Figure 11d) was used to characterize cysteine-dependent DUBs and Ubl proteases.^381^ DUBs cleave the isopeptide or peptide bond C-terminal of Ub G76; therefore, BprY was site-specifically incorporated at position 77 using a previously evolved *Mm*PylRS variant^382^ to achieve ideal proximity to DUB active site nucleophiles. In addition, a G76V mutation was introduced into BprY-bearing Ub to render the probe stable toward DUB cleavage. Western blot and tandem MS analysis confirmed *in vitro* crosslinking with the active site cysteine of the DUB UCHL3. Interestingly, crosslinking to noncysteine nucleophilic residues such as serine, threonine, and histidine within the active center of UCHL3 was also observed, indicating that the Ub-BprY probe could also be used beyond the scope of cysteine-dependent DUBs. Indeed, expression of strep-tagged wt Ub or Ub-BprY in HEK293T cells followed by immunoprecipitation and proteomic analysis revealed the enrichment of more than 2000 proteins, including several known DUBs and Ub interactors, by Ub-BprY. The validity and biological relevance of hits that did not belong to the 57 identified known DUBs or the 18 identified other known Ub interactors were unfortunately not further discussed in the study. Nevertheless, the data set could serve as basis for further investigations. The same applies to the application of BprY-bearing diUb, UFM1, and ISG15 probes that were successfully expressed and crosslinked to a respective deconjugating enzyme in proof-of-concept experiments but not further investigated in mammalian cells.^381^ A different approach for studying the Ub system with proximity-triggered crosslinking ncAAs focused on capturing the transient interaction between Ub-conjugating enzymes and their substrates.^141^ The yeast E2 enzyme UbcH5C and its human homologue UBE2D3 ubiquitylate PCNA at K164. Site-specific incorporation of BprY or its alkyne-bearing analog EB3 at position 164 (or also adjacent positions 163 and 165) within PCNA led to a crosslink with C85 of UBE2D3 *in vitro* and when both proteins were coexpressed in *E. coli* cells.^141^ The versatility of BprY for studying the ubiquitin system was further demonstrated in a study that elucidated the interaction of the bacterial effector protein NleE with the proteasomal subunit PSMD10. In brief, incorporation of BprY into different positions of NleE in HEK293T cells led to proximity-triggered crosslinking with PSMD10. These results contributed to a better understanding of the protein–protein interaction and the role of NleE in the escape of pathogens from autophagy.^383^ Furthermore, BprY was incorporated into SUMO2 to map binding interfaces with SIMs and to probe SUMO2 interaction partners in HEK293T cells.^384^

Regarding aryl fluorosulfate-bearing ncAAs that are able to engage in SuFEx chemistries with nearby nucleophiles, proof-of-principle studies have confirmed the *in cellulo* covalent crosslinking of affibody-Z-protein interfaces when both proteins were overexpressed in *E. coli* or mammalian cells,^379^ as well as the generation of covalently stabilized nanobody conjugates.^385^ As their crosslinking selectivities toward canonical amino acids seem to be complementary to bromoalkane-bearing ncAAs, SuFEx-compatible ncAAs (Figure 11e) could present interesting tools to study interactions in the Ub/Ubl system, but so far only intraprotein crosslinking within Ub bearing FSK at a specific site has been shown.^380^ In conclusion, photocrosslinkers as well as chemical crosslinkers introduced into Ub and SUMO have started to become interesting tools for the identification of writers, readers, and erasers of the Ub system, as well as for their detailed downstream characterization.

### 4.2 A Genetically Encoded Fluorescent DUB Probe

The toolbox of genetically encoded Ub probes, however, is not limited to the incorporation of crosslinking ncAAs. In a recent study, Peng and co-workers showed the site-specific incorporation of two novel coumarin-based fluorescent ncAAs, ACouK and AFCouK (Figure 11f), into proteins in *E. coli* as well as HEK293T cells using a *Mm*PylRS variant. Both ncAAs can form FRET pairs with GFP, which paved the way for the first generation of fully genetically encoded fluorescent DUB probes.^386^ Incubation of a recombinantly expressed fusion construct composed of GFP fused C-terminally to Ub(Y59AFCouK) with the DUB USP7 showed a loss of the FRET signal and an increase in AFCouK emission *in vitro* upon cleavage of the fusion construct between GFP and Ub (Figure 11f). Furthermore, a specific linear diUb probe linked to GFP was developed as a probe for OTULIN, a DUB specific for linearly linked polyUbs. The usefulness of the probes was demonstrated in a DUB inhibitor screening assay with 40 commercially available DUB inhibitors. The results revealed a previously undescribed activity of the UCHL3 inhibitor TCID and the USP30 inhibitor 18 against OTULIN, with IC_50_ values in the nano- to micromolar range. This finding was especially interesting as inhibitors for OTULIN had not been described before. While the authors did not address that the introduction of a large ncAA at position Y59 of Ub could alter the structure of Ub by interfering with the Y59-E51 loop, this could be of interest in future studies on the interactome of genetically encoded fluorescent DUB probes.

## 5 Conclusion and Future Challenges

The comprehensive exploration of a system as multifaceted and versatile as the Ub/Ubl code demands an array of tools capable of tackling its complexity from various angles, drawing on the interdisciplinary expertise of researchers from diverse scientific backgrounds. In this Review, we provide a thorough examination of recent advancements in the application of GCE techniques, which have become essential tools for elucidating the Ub/Ubl code. We highlight GCE approaches for creating Ub/Ubl-POI conjugates with defined linkages. CuAAC, oxime, and thiazolidine ligation yield Ub-POI conjugates with artificial linkages, benefiting applications that demand recalcitrance toward hydrolysis by DUBs, while GOPAL and NCL generate isopeptide-linked conjugates and are ideal if downstream applications call for native linkages between Ub/Ubl and target proteins. These approaches represent proven tools for studying ubiquitylation and have been used for the elucidation and validation of structural features of differently linked diUb variants and Ub chains, as well as for assessing interactomes of Ub/Ubl-conjugates and for profiling the activity of DUBs in cell lysates. As most of these approaches work under conditions that are not applicable to sensitive proteins (*e.g.*, acidic pH, millimolar Cu(I) concentrations, and metal-dependent desulfurization conditions) and therefore require protein refolding, the scope of accessible Ub/Ubl-POI conjugates is somewhat limited. Chemoenzymatic approaches such as sortylation and *Oa*AEP1-mediated ligation that generate isopeptide-linked conjugates offer the added advantage of allowing the ubiquitylation of nonrefoldable proteins under native conditions. Sortylation is so far the only method that allows for triggered and site-specific ubiquitylation and SUMOylation in living cells and is not limited to *in vitro* use. Apart from generating Ub/Ubl-POI conjugates, GCE-based methods have also enabled the generation of homogeneously site-specifically acetylated, phosphorylated, or succinylated Ub/Ubl variants to study the influence of these small-molecule PTMs. Last, but not least, site-specifically introduced crosslinker or fluorescent moieties complement the GCE-based toolkit to study the Ub code by providing means to elucidate PPIs and to profile enzyme activities.

Despite the value of these methods, challenges remain. Current GCE-based methods for making Ub/Ubl-POI conjugates often depend on harsh conditions, limiting their applicability to simple, refoldable proteins. As discussed, chemoenzymatic methods can address these limitations, and the discovery and evolution of new transpeptidases to enable completely scarless natively linked Ub/Ubl-POI conjugates or alternatively the design of novel amide-bond forming bioorthogonal reactions may further expand the range of accessible conjugates.^387^ As sortylation represents so far the only available method to artificially trigger site-specific ubiquitylation in living cells, its potential to study the consequences of individual ubiquitylation events *in cellulo* should be harnessed in future applications.

Apart from lysine ubiquitylation, recent discoveries have revealed the important roles of serine and threonine ubiquitylation in endoplasmic reticulum-associated degradation, immune signaling, and neuronal processes.^16^ While an ester-linked Ub-α-globin conjugate was previously accessed via total chemical synthesis and was used for profiling DUBs,^259^ GCE-based tools to study ester-linked Ub-POI conjugates are so far missing, and adaptation of the bioorthogonal or chemoenzymatic approaches described herein to access such noncanonical Ub-POI conjugates would be highly valuable. Similarly, the majority of current studies and tools are primarily centered on Ub, highlighting the potential for future GCE-based studies to explore other Ubls. Regarding the study of small-molecule PTMs and their impact on ubiquitylation events, considerable progress has been achieved in studying acetylation and phosphorylation, but clever approaches for precisely modifying Ub/Ubl with complex PTMs recently identified, such as arginine phosphoribosylation and ADP ribosylation,^16^ are urgently needed. Similarly, the potential of Ub/Ubl-based probes for mapping reader proteins and profiling writer and eraser enzymes has not yet been fully realized. There is a need for crosslinking probes that better mimic transient E3:E2:Ub-POI intermediates to elucidate the structures of these complexes and delineate their mechanistic principles. Furthermore, design of novel crosslinking ncAAs and their site-specific encoding via GCE may enable DUB and HECT-based E3 specificities to be profiled in living cells rather than being confined to *in vitro* or lysate-based studies. This may facilitate unprecedented insights into linkage and substrate specificities of writer and eraser enzymes and may allow profiling of their activities as a function of subcellular localization. Smart combination of such technologies with advances in proteomic methods may furthermore allow system-level interrogation of the whole Ub system.

It is inherent to the nature of GCE that protein production yields are typically lower than those obtained for the expression of wt proteins. This difference depends on the ncAA itself, its cell-permeability and metabolic stability, the used aaRS/tRNA pair, the POI, the position of the suppressed codon within the POI, and the expression system and can be a marginal or, rarely, a real limitation for envisioned downstream applications. Recent efforts in advancing GCE technologies, such as the development of new or improved orthogonal aaRS/tRNA pairs^97,98,100,101^ and engineered expression hosts,^110^ aim to overcome this limitation to make use of unmined potential for future studies that, for example, lies in the efficient generation of bifunctional Ub/Ubl variants displaying two different ncAAs by dual suppression. This will allow the study of PTM crosstalk or enable the assessment of interactomes of site-specifically modified Ub/Ubl variants by incorporating a crosslinker ncAA and a PTM-bearing ncAA into the same Ub/Ubl building block. Furthermore, combining established GCE-based methods will allow the generation of even more complex polyUb/Ubl-POI topologies that carry a site-specific crosslinker moiety for the identification of receptor and effector proteins that translate these complex Ub/Ubl patterns into a specific outcome for the modified protein.

Ultimately, we are convinced that GCE-based tools offer promising avenues for unraveling the complexity of the Ub code. Continued advances in the field, combined with creative chemical probe design and advancements in protein engineering, will help overcome existing challenges to provide deeper biological insights beyond proof-of-principle studies. Importantly, GCE tools should be made available and accessible to researchers from different disciplines, fostering collaborations between tool-developers and tool-appliers to understand this most peculiar PTM in more detail and to spur biological discovery.

## Abbreviations

2DPBA: 2-(Diphenylphosphino)benzoic acid
AAA: ATPase associated with a variety of cellular activities
aaRS: Aminoacyl-tRNA synthetase
ABP: Activity-based probe
AcK: *N*ε-Acetyl-l-lysine
Aha: l-Azidohomoalanine
Alloc: Allyloxycarbonyl
AMP: Adenosine monophosphate
ASHA: Acylsilanes with hydroxylamines
ATP: Adenosine triphosphate
AzF: *p*-Azidophenylalanine
AznL: Azidonorleucine
Boc: *tert*-butoxycarbonyl
Boc-εOK: *N*ε-(*tert*-Butyloxycarbonyl)aminooxy-l-lysine
BocK: *N*ε-(*tert*-Butoxycarbonyl)-l-lysine
Bpa: *p*-Benzoyl-l-phenylalanine
BprY: *O*-(3-Bromopropyl)-tyrosine
BRCA1: Breast cancer type 1 susceptibility protein
CaM: Calmodulin
Cbz: Benzyloxycarbonyl
Cbz-OSu: *N*-(Benzyloxycarbonyloxy)succinimide
cmF: *p*-Carboxymethyl-l-phenylalanine
CuAAC: Cu(I)-catalyzed 1,3-dipolar azide–alkyne cycloaddition
Daxx: Death domain-associated protein 6
DUB: Deubiquitylating enzyme
Dvl2: Dishevelled 2
*E. coli*: *Escherichia coli*
EPL: Expressed protein ligation
FRET: Förster resonance energy transfer
GCE: Genetic code expansion
GFP: Green fluorescent protein
GOPAL: Genetically encoded orthogonal protection and activated ligation
HECT: Homologous to the E6-AP carboxyl terminus
HEK293T: Human embryonic kidney 293 cells (containing gene for SV40 large T antigen)
hHR23A: Human homologue of yeast Rad23a
ISG15: Product of interferon (IFN)-stimulated gene 15
KAHA: α-Ketoacid-hydroxylamine
KAT: Potassium acyltrifluoroborates
KetoK: (*S*)-2-Amino-8-oxononanoic acid
*Ma*: *Methanomethylophilus alvus*
*Mb*: *Methanosarcina barkeri*
MetRS: Methionyl-tRNA synthetase
MIDA: *N*-Methyliminodiacetyl acylboronates
MINDY: Ubiquitin (MIU)-containing novel DUB family
*Mj*: *Methanocaldococcus jannaschi*
*Mm*: *Methanosarcina mazei*
MMTS: Methanethiosulfonate
MYCBP2: MYC binding protein 2
ncAA: Noncanonical amino acid
NCL: Native chemical ligation
NEDD8: Neural-precursor-cell-expressed developmentally down-regulated 8
NZF: Npl4 zinc finger
*Oa*AEP1: *Oldenlandia affinis* asparaginyl endopeptidase 1
OMM: Outer mitochondrial membrane
OTU: Ovarian tumor protease
OTUB1: Otubain 1
OTUB2: Otubain 2
*p*-nitroCbz-δThioK: δ-Thiol-*N*ε-(*p*-nitrocarbobenzyloxy)-l-lysine
p53: Cellular tumor antigen p53
PARP1: Poly(ADP-ribose) polymerase 1
paY: Phosphoramidate derivative of pY
PCNA: Proliferating cell nuclear antigen
PIAS1: SUMO E3 ligase PIAS1
PINK1: Serine/threonine-protein kinase PINK1
PlK: *N*ε-(propargyloxycarbonyl)-l-lysine
PML: Promyelocytic leukemia protein
Pmp: 4-Phosphomethyl-l-phenylalanine
POI: Protein of interest
Pol: DNA polymerase
PPI: Protein–protein interaction
PTM: Post-translational modification
pY: Phosphotyrosine
Pyl: Pyrrolysine
PylRS: Pyrrolysyl-tRNA synthetase
RanBP2: Ran-binding protein 2
RanGAP: Ran GTPase-activating protein
Rap80: Receptor associated protein 80
RBR: RING-between-RING
RCR: RING-Cys-relay
RF1: Release factor 1
RFC: Replication factor C
RING: Really Interesting New Gene
RNF213: Ring finger protein 213
Sec: Selenocysteine
SDS: Sodium dodecyl sulfate
SIRT1: Sirtuin 1
SIRT2: Sirtuin 2
Sp100: Nuclear autoantigen Sp-100
Srt2A: Sortase 2A
Srt5M: Sortase 5M
SUMO: Small ubiquitin-related modifier
TAcK: *N*ε-Thioacetyl-l-lysine
TFA: Trifluoroacetic acid
TFAcK: *N*ε-Trifluoroacetyl-l-lysine
ThzK: *N*ε-Thiaprolyl-l-lysine
TyrRS: Tyrosyl-tRNA synthetase
Ub: Ubiquitin
UBA: Ub-associated domain
UBA1: Ubiquitin-like modifier-activating enzyme 1
UBD: Ubiquitin binding domain
Ubl: Ubiquitin-like proteins
UBZ: Ub-binding zinc finger
UCH: Ubiquitin C-terminal hydrolase
UEV1A: Ubiquitin-conjugating enzyme E2 variant 1A
UIM: Ub-interacting motif
USP: Ubiquitin-specific protease
USP2: Ubiquitin-specific protease 2
USP5: Ubiquitin-specific protease 5
wt: Wild type
ZUP1: Zinc finger containing ubiquitin peptidase 1
